# Supplementary material for: Millisecond Coupling of Local Field Potentials to Synaptic Currents in the Awake Visual Cortex
Source: Neuron. 2016 Apr 6;90(1):35–42. doi: 10.1016/j.neuron.2016.02.034 (PMC4826437; doi:10.1016/j.neuron.2016.02.034)
Supplement: Document S1. Supplemental Experimental Procedures and Figures S1–S5 [file mmc1.pdf]

Neuron, Volume *90*

Supplemental Information

Millisecond Coupling of Local Field Potentials to Synaptic Currents in the Awake Visual Cortex

Bilal Haider, David P.A. Schulz, Michael Häusser, and Matteo Carandini

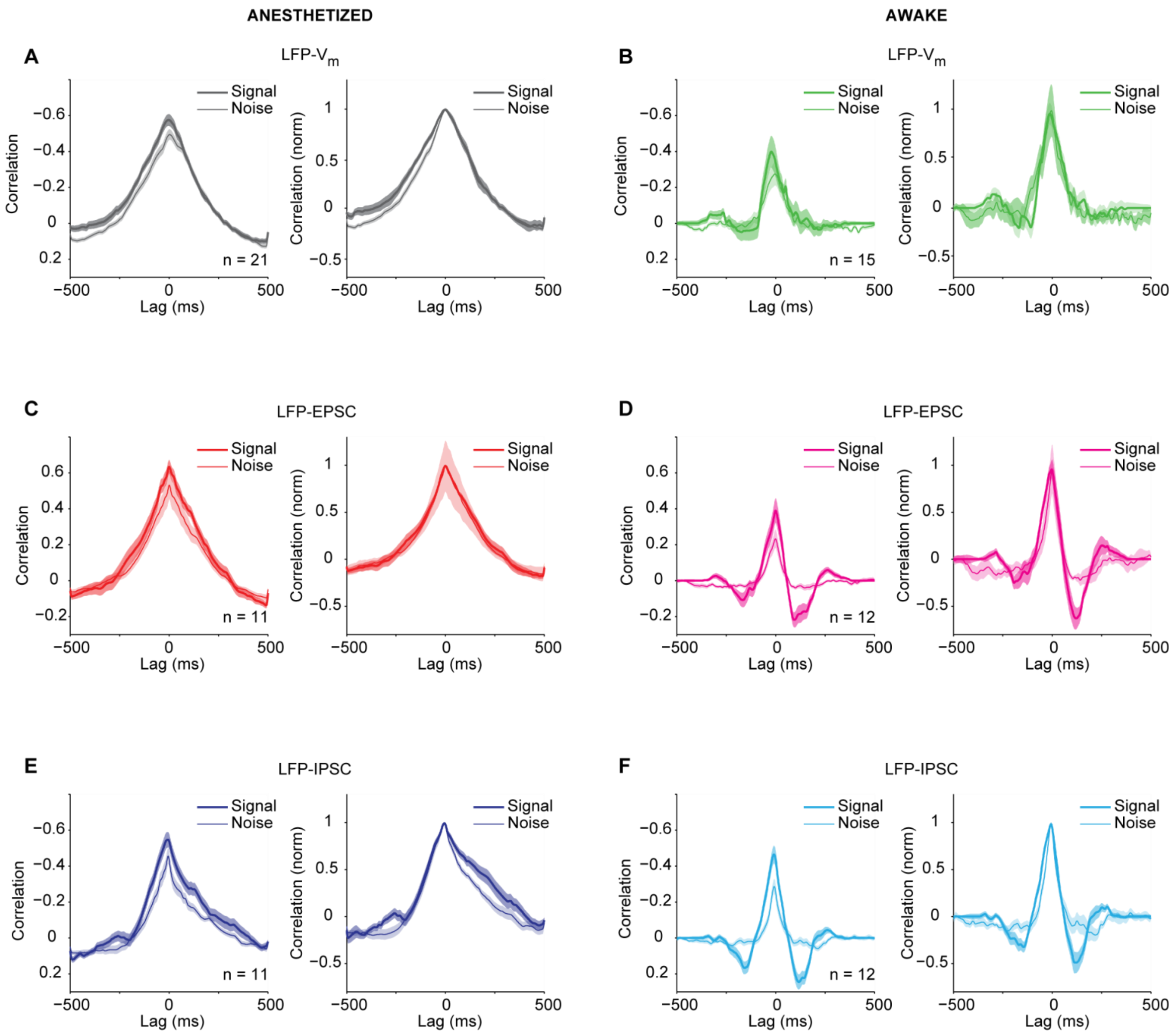

FIGURE S1 (related to Figure 1). Time course of anesthetized and awake signal and noise correlations.

A. LFP-V_m signal (darker) and noise (lighter) correlations during anesthesia (left), and signal and noise correlations normalized to peak (right). Note broad timescales. Noise correlations calculated by subtracting average stimulus response from every individual trial. Traces show median ± SEM (shaded).

B. Same as in A, for awake LFP- V_m signal and noise correlations.

C. Same as in A, for anesthetized LFP-EPSC signal and noise correlations.

D. Same as in A, for awake LFP-EPSC signal and noise correlations.

E. Same as in A, for anesthetized LFP-IPSC signal and noise correlations.

F. Same as in A, for awake LFP-IPSC signal and noise correlations.

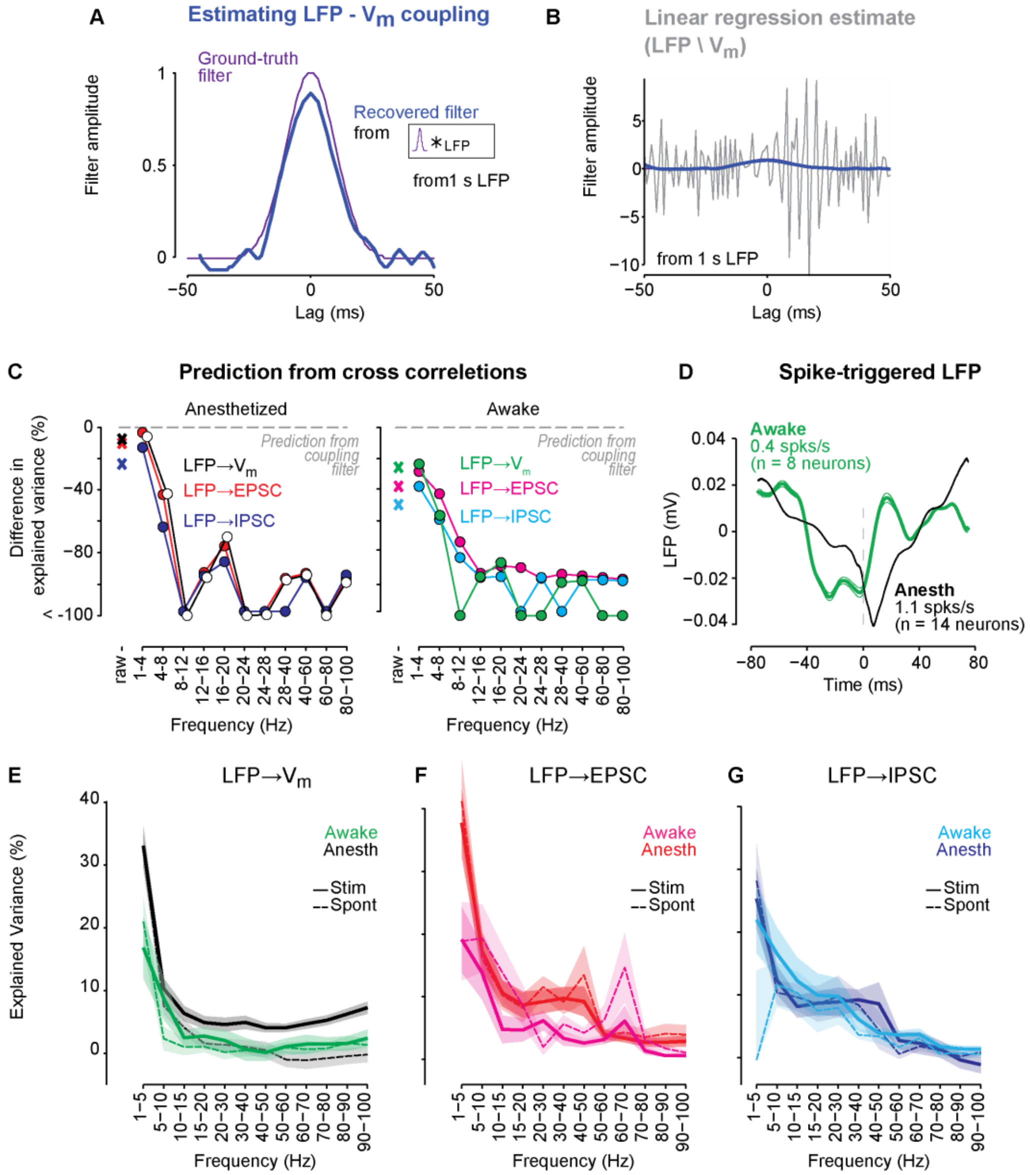

FIGURE S2 (related to Figures 1 and 2). Regularized filter estimation accurately recovers ground-truth coupling.

A. LFP traces (1 s snippets) were convolved with a ground-truth filter (Normalized Gaussian, violet) and contaminated with additive Gaussian noise to produce 'fake' V_m. Filter estimation procedure was then used to recover optimal coupling of LFP to 'fake' V_m (blue). Note similar shapes of ground-truth filter and recovered filter.

B. Filter estimated from unregularized linear regression (grey) on same data set fails to recover ground-truth coupling. The highly irregular shape of the estimated filter is due to overfitting high frequencies.

C. Predictions from cross correlations were substantially worse than predictions from coupling filters. Filters shaped exactly like cross correlations (for each individual paired recording, during visual stimulation) were used to predict intracellular traces from the LFP. We compared the variance explained by these predictions to variance explained by optimal coupling filters (% difference). Cross correlation predicts most poorly during wakefulness (Right: raw V_m (green) 26% worse, raw EPSCs (magenta) 38% worse, raw IPSCs (cyan) 50% worse). Cross correlations failed to predict information in higher frequencies (circles). Same population of neurons as in Figures 2 and 3.

D. Stimulus-evoked spike-triggered LFP (stLFP) during wakefulness (green) is narrower and smaller than during anesthesia (black). Mean ± SEM.

E-G. Explained variance as a function of frequency for LFP-Vm, LFP-EPSC, and LFP-IPSC coupling. Mean ± SEM.

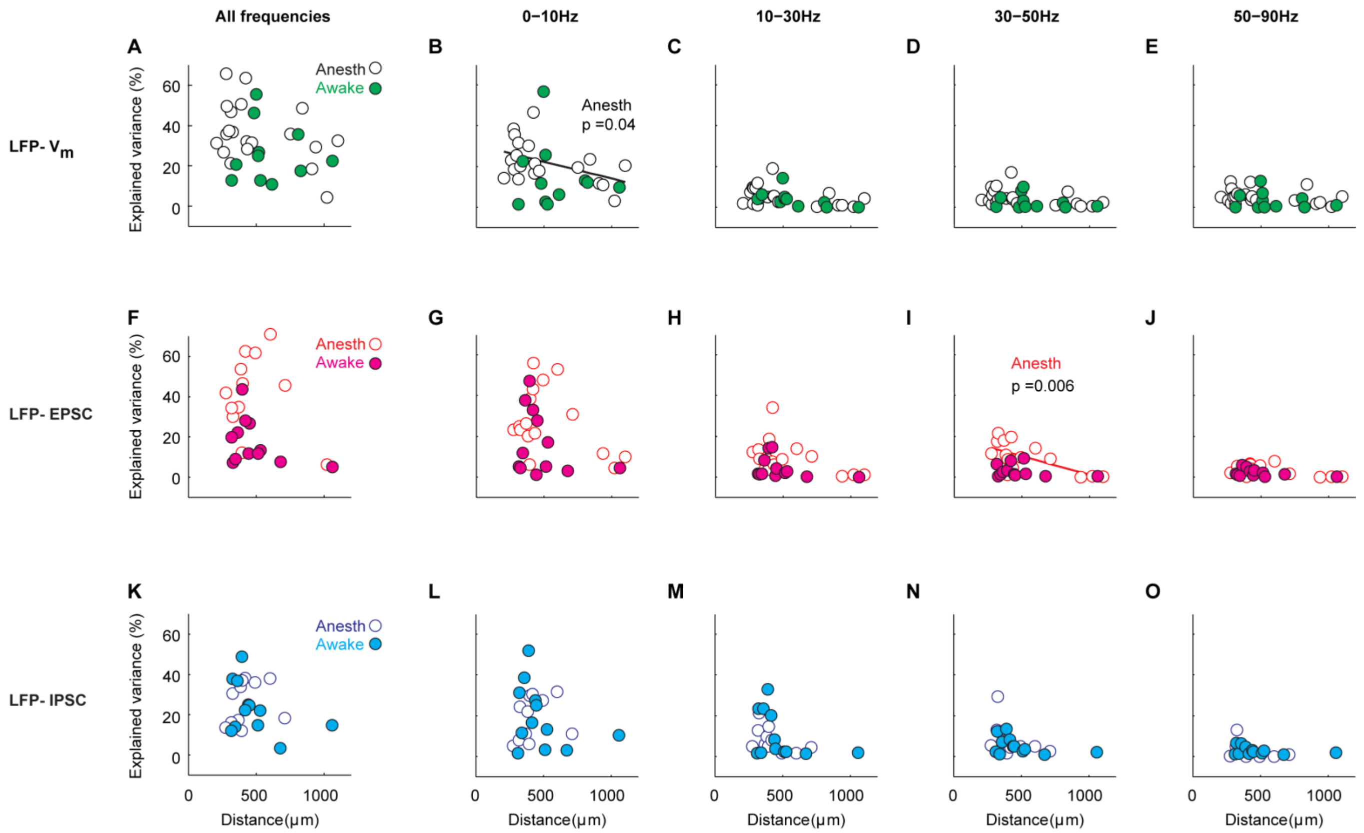

FIGURE S3 (Related to Figures 2 and 3). Explained variance as a function of frequency band and separation distance.

A-E. Stimulus-evoked LFP-V_m predictions during anesthetized and awake states. **A**, Explained variance of raw signals, as a function of separation between LFP and single neuron recording. **B**, Explained variance for signals filtered < 10Hz. Significant correlation with distance during anesthesia. **C**, Explained variance for signals filtered from 10 - 30Hz. **D**, Explained variance for signals filtered from 30 - 50Hz. **E**, Explained variance for signals filtered from 50 - 90Hz.

F-J. Stimulus-evoked LFP-EPSC predictions during anesthetized and awake states. **F**, Explained variance of raw signals, as a function of separation between LFP and single neuron recording **G**, Explained variance for signals filtered < 10Hz; **H**, Explained variance for signals filtered from 10 - 30Hz. **I.** Explained variance for signals filtered from 30 - 50Hz. Significant correlation during anesthesia. **J**, Explained variance for signals filtered from 50 - 90Hz.

K-O. Stimulus-evoked LFP-IPSC predictions during anesthetized and awake states. **K**, Explained variance of raw signals, as a function of separation between LFP and single neuron recording. **L**, Explained variance for signals filtered < 10Hz. **M**, Explained variance for signals filtered from 10 - 30Hz. **N,** Explained variance for signals filtered from 30 - 50Hz. **O**, Explained variance for signals filtered from 50 - 90Hz.

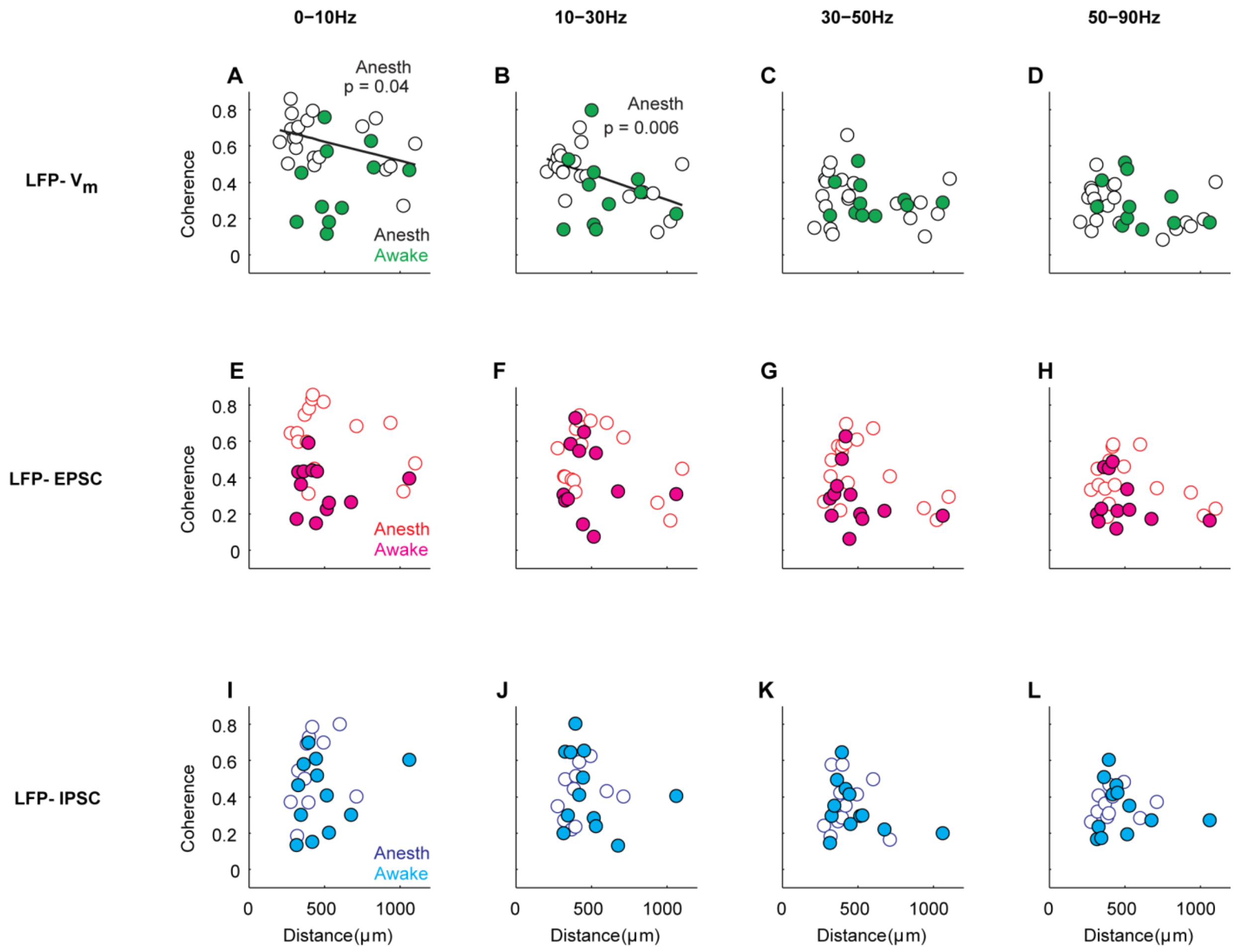

FIGURE S4 (Related to Figures 2 and 3). Coherence as a function of frequency band and separation distance.

A-D. Stimulus-evoked LFP-V_m coherence during anesthetized and awake states. Mean coherence plotted within band (see Figure S5 for coherence plots). **A**, Mean coherence < 10Hz. Significant correlation with distance during anesthesia. **B**, Coherence for 10 - 30Hz. Significant correlation with distance during anesthesia. **C**, Mean coherence from 30 - 50Hz. **D**, Mean coherence from 50 - 90Hz.

E-H. Stimulus-evoked LFP-EPSC coherence during anesthetized and awake states. Mean coherence plotted within band (see Figure S5 for coherence plots). **E**, Mean coherence < 10Hz. **F**, Coherence for 10 - 30Hz. **G**, Mean coherence from 30 - 50Hz. **H**, Mean coherence from 50 - 90Hz.

I-L. Stimulus-evoked LFP-V_m coherence during anesthetized and awake states. Mean coherence plotted within band (see Figure S5 for coherence plots). **I**, Mean coherence < 10Hz. **J**, Coherence for 10 - 30Hz. **K**, Mean coherence from 30 - 50Hz. **L**, Mean coherence from 50 - 90Hz.

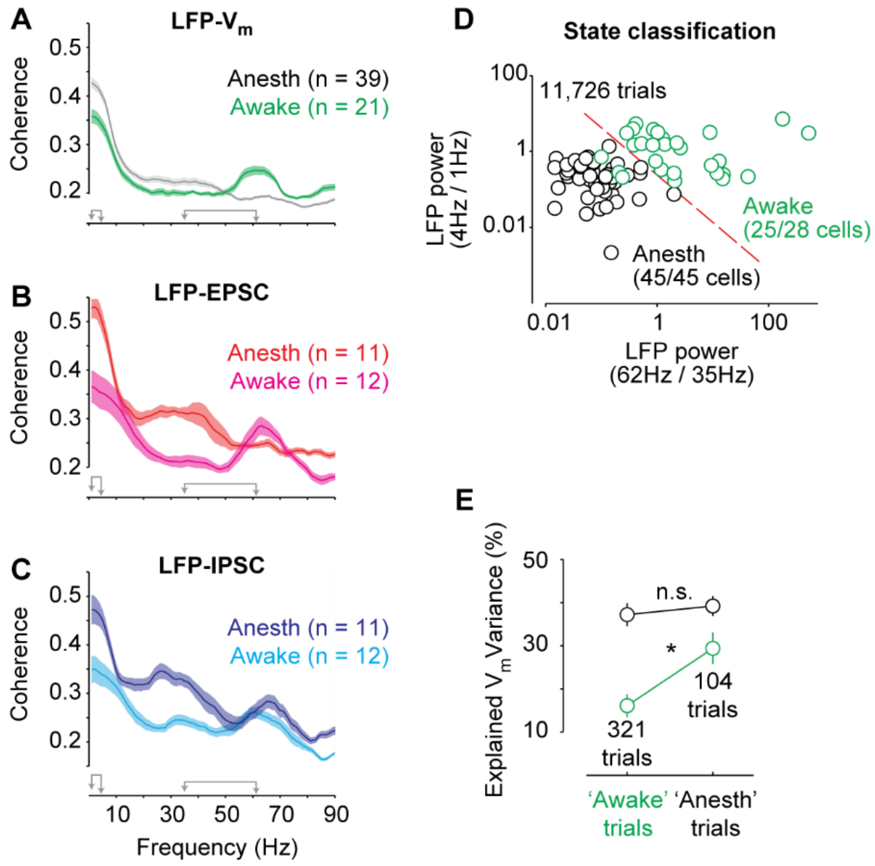

Figure S5 (Related to Figure 3). LFP features accurately distinguish anesthetized and awake states.

A. We wondered whether the differences in LFP coupling between anesthesia and wakefulness could arise from differences in the LFP signal itself. We observed clear differences in anesthetized and awake LFP spectral power, and LFP coherence with single neuron V_m across all paired recordings (anesthetized, black, n = 39; awake, green, n = 21). Arrows, 1-4 Hz and 35-62 Hz.

B. Spontaneous LFP-EPSC coherence (anesthetized, red, n = 11; awake, magenta, n = 12).

C. Spontaneous LFP-IPSC coherence (anesthetized, blue, n = 11; awake, cyan, n = 12).

D. We built a classifier that used LFP features to assign experiments to anesthetized and awake groups. This procedure segregated experiments with 96% accuracy (n = 73 recordings). Ratios of high-frequency (62 / 35 Hz) vs. low frequency (4 Hz / 1 Hz) LFP power accurately classify experimental conditions (n = 45/45 anesthetized recordings; n = 25/28 awake recordings correctly identified; 11,726 total trials). Power was calculated on individual trials, then averaged across trials (within recording) before taking log power ratios.

E. We then used the classifier to identify single trials where awake LFP activity was classified as anesthetized (and vice versa). On single trials where awake spontaneous activity displayed 'anesthetized' LFP power ratios (right, 104/425 individual trials), spontaneous V_m was in fact significantly more predictable than correctly classified awake trials (29.4 ± 3.6% vs. 16.1 ± 2.7%, $p < 0.01$ Wilcoxon rank sum test). No significant difference was observed across trial types during anesthesia.

These results show that cortical state fluctuations can be accurately segregated from the LFP alone, and confirm that cortical state fluctuations drive large trial-by-trial differences in the LFP and its coupling to single neurons.

Supplemental Experimental Procedures

Recordings

All procedures were in accordance with the Animals (Scientific Procedures) Act 1986, UK.

We made simultaneous LFP recordings and whole-cell patch-clamp recordings in L2/3 of mouse primary visual cortex (V1). LFP recordings were performed with glass patch pipettes filled with artificial cerebrospinal fluid (aCSF, 135mM NaCl, 5.4mM KCl, 5mM HEPES, 1mM $MgCl_2$ and1.8mM $CaCl_2$; pH 7.3).

Whole-cell patch-clamp recordings of V_m were performed with a K^+ gluconate internal solution (135mM potassium gluconate, 6mM KCl, 10mM HEPES, 4mM MgATP, 0.3mM Na_3GTP, 0.1mM EGTA and 8mM phosphocreatine, pH adjusted to 7.3 with KOH, 290–295 mOsM). For voltage clamp recordings of EPSCs (recorded near -80 mV) and IPSCs (recorded near +20 mV), potassium gluconate was substituted with 140mM cesium methanesulphonate, and QX-314 (0.5mM) and tetraethylammonium (TEA) (5mM) were included to block voltage-gated Na^+ and K^+ conductances.

The typical separation between our LFP and intracellular electrodes was ~0.5 mm (Anesthetized: 0.46 ± 0.02 mm; Awake: 0.53 ± 0.02 mm, mean ± sem), with a range of 0.2-1.1 mm, comparable to previous studies of LFP and subthreshold activity in awake cortex (Okun et al, 2010; Poulet and Petersen, 2008)

Most anesthetized recordings were performed under 0.5% – 1% isoflurane (Vm: 28/39 spontaneous, 18/21 stimulus-evoked; PSCs: 7/11) or urethane (1.5 g per kg, 10% w/v solution administered IP), plus chlorprothixene (10^{-5} mg per kg) in both cases. Awake recordings were performed in drug-free mice that were habituated to head-fixation over several days. Access resistance (in MΩ) was comparable across states, and was partially compensated during voltage clamp recordings (Anesth. current clamp: 40 ± 3; Awake current clamp: 38 ± 6; Anesth. voltage clamp: 36 ± 3 (15 ± 2 after compensation); Awake voltage clamp: 38 ± 4 (14 ± 2 after compensation).

Visual stimulation

Randomly chosen full contrast vertical black or white bars (9 degrees wide) were briefly flashed (100 ms duration), one at a time, in a randomly chosen spatial location on an LCD monitor spanning ± 45 degrees of visual space. Interstimulus intervals were 0.3-1.5 s. All recordings were performed in monocular V1 contralateral to the stimulated eye, with the receptive field of the recording at the center of the monitor (see Haider et al. 2013). Spontaneous activity was recorded in the presence of an isoluminant grey screen. Since both LFP and sub-threshold synaptic activity were elicited from large regions of visual space, we combined activity from all spatial locations for analysis of evoked activity.

Data processing

All signals were acquired at 20 kHz. LFP and PSCs were hardware filtered prior to digitization (LFP: 0.01-200 Hz; PSCs: < 2 kHz). The recordings of EPSCs and IPSCs were matched within neurons, performed sequentially, and with the same physical filter characteristics (of electrodes, series resistance, and capacitance) for paired EPSCs and IPSCs, across all individual experiments. Action potentials were digitally removed from V_m traces. The coherence between LFP and intracellular activity was computed using the Chronux toolbox (time-bandwidth NW=10, K=21 tapers) (Bokil et al., 2010).

Filter estimation

We first subtracted the mean across time from all LFP and V_m, EPSC, or IPSC traces. For the purposes of filter estimation during visual stimulation, we included the entire visual response (which lasted up to 1.5 second during anesthesia). We then estimated a filter constrained to be smooth in time via regularized linear regression. The algorithm works with the following quantities, where N is the number of samples in time:

- The LFP $\vec{u} = (u_1, \ldots, u_N)$ serving as input to the linear model.
- A trace (V_m / EPSC / IPSC) $\vec{y} = (y_1, \ldots, y_N)$ serving as output to be fitted.
- A set of time lags $\tau_1, \ldots, \tau_K$ spanning the filter $\vec{w} = \left(w_{\tau_1}, w_{\tau_2}, \ldots, w_{\tau_K}\right)$ to be estimated.

A so-called “lag matrix” $X = (\vec{x}_1, \ldots, \vec{x}_N)^T$ is formed by stacking vectors $\vec{x}_t$ which are formed by taking temporally extended snippets of the input $\vec{u}$ such that $\vec{x}_t = \left(u_{t-\tau_1}, \ldots u_{t-\tau_K}\right)$.

A second matrix $C_{\sigma\lambda}$ – the prior covariance matrix - encodes the smoothness structure imposed on the optimal filter $\vec{w}_{\sigma\lambda}$, which – within the framework of regularized linear regression – is estimated as

$$\vec{w}_{\sigma\lambda} = (C_{\sigma\lambda}^{-1} + X^T X)^{-1} X^T \vec{y} \qquad (1)$$

The regularization structure is parameterized by a pair of numbers, the prior variance σ^2 and the smoothness λ, such that the prior covariance between filter entries w_i and w_j is given by $C_{\sigma\lambda}(w_i, w_j) = \sigma^2 \times \rho(w_i, w_j)$ where $\rho(w_i, w_j)$ is the prior correlation between filter weights w_i and w_j .

We chose an exponential correlation function in time, resulting in the following formula for the correlation:

$$\rho(w_i, w_j) = exp\left(-\frac{|\tau_i - \tau_j|}{\lambda}\right), \qquad (2)$$

where τ_i, τ_j are the filter lags of the weights.

When λ is small, the correlation of filter points nearby in time is also small. For example, this means that the filter value w_1 at time-lag τ_i=1 ms is largely independent from the value w_2 at a nearby point in time, such as τ_j=2 ms. Thus, a small λ captures our assumptions that *a priori* the filter $\vec{w}$ shows little smoothness in time.

However, when λ is large, then the correlation of nearby filter points is also large, meaning that *a priori* the filter values w_i and w_j should be similar, resulting in a temporal filter that looks smooth in time. Assuming that filters have a certain smoothness in time is reasonable, since single neurons and populations cannot respond arbitrarily fast. Furthermore, the smoothness prior prevents the algorithm from overfitting high-frequency noise which contains no signal (Suppl. Fig. 1).

We estimated the optimal hyperparameters $(\sigma, \lambda)_{opt}$ by performing gradient descent on the 10-fold cross-validation error. A given dataset is split into 10 disjoint parts, then filters are trained on 9 parts with the current prior covariance matrix $C_{\sigma\lambda}$. The estimated filter is used to predict the response on the omitted (test) set. The gradient is computed, iterated for all 10 parts, and all 10 gradients and errors are summed to search for the optimal hyperparameters $(\sigma, \lambda)_{opt}$ at which the cross-validation error is minimal. At that minimum, the trade-off between capturing the signal and avoiding overfitting to the noise is optimal.

The optimal hyperparameters averaged across cells and conditions were σ_{Vm} = 1.1, λ_{Vm} = 55 ms; σ_{EPSC} = 1.6, λ_{EPSC} = 50 ms; σ_{IPSC} = 3.2, λ_{IPSC} = 60 ms. The population of all filters is shown in Figs. 1 and 2 (mean ± SEM).

We did not use a separate test set that remained untouched throughout the analysis; a final test set is usually only employed when doing model comparisons, to determine which model performs best. We did not ask that question – we only used a single model.

We found the fitted hyperparameters to be similar across subsets of the data in the same condition. More specifically, when we determined the best hyperparameters for each of the 10 held-out sets, we found that they lie close to each other, strongly suggesting that no overfitting occurred.

In general, the risk of overfitting the hyperparameters is small because within each condition (e.g. spontaneous LFP-Vm, awake) we only fit two quantities, the variance σ^2 and the smoothness λ to the entire data set: tens of neurons with thousands of individual trials. When a vast amount of data is available to constrain just 2 numbers, the risk of overfitting is negligible.

Role of Autocorrelations

Slow autocorrelations of the LFP are the dominating factor in determining the shape of cross correlations. To obtain the underlying coupling filters, we need to correct for this autocorrelation. This correction, however, is only possible through regularization: there is too little energy at high frequencies in the autocorrelation, and without regularization this leads to noisy estimates.

To see this, consider first the case without regularization:

$$\vec{w}_{\text{unregularized}} = (X^T X)^{-1} X^T \vec{y} \tag{3}$$

where X is the LFP lag matrix, whose row vectors consist of time-shifted LFP traces. The matrix $X^T X$ is the autocorrelation matrix of the LFP, and $X^T \vec{y}$ is the cross-correlation of LFP with the signal of interest, $\vec{y}$.

To see the role of different frequencies in this estimation, let's turn to Fourier space – here we estimate the Fourier transform of the coupling filter by dividing the Fourier transform of the cross-correlation $X^T \vec{y}$ by the Fourier transform of the autocorrelation $X^T X$. If the latter is dominated by slow frequencies, its energy at high frequencies is small. Dividing by small and noisy quantities yields large and even noisier quantities.

This is indeed what we found when we followed this procedure: the filter estimated from unregularized linear regression is noisy, and does not recover ground-truth coupling because of high frequency noise (Fig. S2B). This reasoning reflects the fact that the autocorrelation contains little power at high frequencies and has its largest power at low frequencies.

The key contribution of the regularization procedure (Eq. 1) is to add just about enough energy $C_{\sigma\lambda}^{-1}$ at different frequencies of the autocorrelation function to reduce the impact of the noise when dividing by small noisy quantities. The regularization procedure (Eq. 1) is equivalent to filtering the cross-correlation $X^T \vec{y}$ by a filter F = $(C_{\sigma\lambda}^{-1} + X^T X)^{-1}$. But the filter F is not just any highpass filter: it is the optimal filter. We find it through a linear model and its associated squared error, which serves as a cost function and depends on hyperparameters σ, λ. If we only used the raw cross-correlation, we would have no such principled method of determining the frequency characteristics of the optimal filter for each particular condition.

References

Bokil, H., Andrews, P., Kulkarni, J.E., Mehta, S., and Mitra, P.P. (2010). Chronux: a platform for analyzing neural signals. J Neurosci Methods *192*, 146-151.
