# Supplementary material for: Millisecond Coupling of Local Field Potentials to Synaptic Currents in the Awake Visual Cortex
Source: Neuron. 2016 Apr 6;90(1):35–42. doi: 10.1016/j.neuron.2016.02.034 (PMC4826437; doi:10.1016/j.neuron.2016.02.034)
Supplement: Document S2. Article plus Supplemental Information [file mmc2.pdf]

Report

Neuron

Millisecond Coupling of Local Field Potentials to Synaptic Currents in the Awake Visual Cortex

Highlights

- Regularized model reveals fast coupling of LFP and single neurons in mouse V1
- Synaptic currents determine the relationship of LFP to single neurons
- During wakefulness, LFP is coupled to both synaptic excitation and inhibition
- During wakefulness, stimuli increase coupling of LFP to synaptic activity

Authors

Bilal Haider, David P.A. Schulz, Michael Häusser, Matteo Carandini

Correspondence

bilal.haider@bme.gatech.edu

In Brief

Haider et al. show that the local field potential (LFP) can accurately predict synaptic excitation and inhibition in neurons of awake visual cortex. These findings may help interpret the LFP and other macroscopic brain signals, such as EEG and ECoG.

Haider et al., 2016, Neuron *90*, 35–42
April 6, 2016 ©2016 The Authors
http://dx.doi.org/10.1016/j.neuron.2016.02.034

CellPress

Millisecond Coupling of Local Field Potentials to Synaptic Currents in the Awake Visual Cortex

Bilal Haider,[1,3,4,*] David P.A. Schulz,[1,3,5] Michael Häusser,[2] and Matteo Carandini[1]
[1]UCL Institute of Ophthalmology, University College London, London EC1V 9EL, UK
[2]Wolfson Institute for Biomedical Research, University College London, London WC1E 6BT, UK
[3]Co-first author
[4]Present address: Coulter Department of Biomedical Engineering, Georgia Institute of Technology and Emory University, Atlanta, GA 30332, USA
[5]Present address: Group for Neural Theory, École Normale Supérieure, 75005 Paris, France
*Correspondence: bilal.haider@bme.gatech.edu
http://dx.doi.org/10.1016/j.neuron.2016.02.034
This is an open access article under the CC BY license (http://creativecommons.org/licenses/by/4.0/).

SUMMARY

The cortical local field potential (LFP) is a common measure of population activity, but its relationship to synaptic activity in individual neurons is not fully established. This relationship has been typically studied during anesthesia and is obscured by shared slow fluctuations. Here, we used patch-clamp recordings in visual cortex of anesthetized and awake mice to measure intracellular activity; we then applied a simple method to reveal its coupling to the simultaneously recorded LFP. LFP predicted membrane potential as accurately as synaptic currents, indicating a major role for synaptic currents in the relationship between cortical LFP and intracellular activity. During anesthesia, cortical LFP predicted excitation far better than inhibition; during wakefulness, it predicted them equally well, and visual stimulation further enhanced predictions of inhibition. These findings reveal a central role for synaptic currents, and especially inhibition, in the relationship between the subthreshold activity of individual neurons and the cortical LFP during wakefulness.

INTRODUCTION

The aggregate activity of neuronal populations in the cortex produces an electrical signal that can be easily measured from outside the cranium (electroencephalography) or within the cortex (local field potential, LFP). The LFP reflects the activity of local populations (Buzsáki et al., 2012; Katzner et al., 2009; Destexhe et al., 1999), is closely linked to the blood-oxygen signal measured in fMRI (Logothetis et al., 2001), and is an attractive signal for brain-machine interfaces. The current sources and sinks generating the LFP have been well-studied in vitro and under anesthesia; nonetheless, there remain open questions regarding the usefulness of the LFP for inferring synaptic activity of individual neurons in intact, awake cortex.

One open question concerns the relationship of the LFP to intrinsic, nonsynaptic currents in single neurons (Buzsáki et al., 2012). Studies in vitro indicate that intrinsic currents provide a measurable contribution to the LFP in both hippocampus (Jefferys and Haas, 1982) and neocortex (Buzsáki et al., 1988). Large-scale simulations have offered differing estimates of this contribution: some suggest that the LFP mainly reflects synaptic activity (Einevoll et al., 2013; Lindén et al., 2011), and others suggest a dominant role for intrinsic conductances (Reimann et al., 2013). It is not known how the LFP relates to subthreshold activity in single neurons, particularly in the intact and awake cortex.

A second question concerns the relative roles of synaptic excitation and inhibition. The traditional view is that the cortical LFP largely reflects the activity of pyramidal neurons, which are numerous and well-aligned in space (Braitenberg and Schuz, 1991). Indeed, activation of excitatory afferents produces large and localized extracellular current sinks (Mitzdorf and Singer, 1978). Inhibition, conversely, is thought to contribute little to the cortical LFP because inhibitory cells and synapses are fewer, have lower driving force, and are not well-aligned in space (Bartos et al., 2007; Hubbard et al., 1969). However, measurements in vitro have revealed conditions where the LFP reflects inhibitory activity, both in hippocampus (Bazelot et al., 2010; Glickfeld et al., 2009) and in neocortex (Trevelyan, 2009). Moreover, inhibitory currents recorded in vitro and under anesthesia are involved in fast patterns in the LFP (Atallah and Scanziani, 2009; Hasenstaub et al., 2005; Okun and Lampl, 2008; Penttonen et al., 1998; Poo and Isaacson, 2009; Salkoff et al., 2015). It remains unknown how the LFP is related to synaptic excitation and inhibition of individual neurons in the intact, awake cortex.

A third question concerns the effect of sensory stimuli on the relationship between the LFP and synaptic currents. Extracellular recordings show that visual stimulation reduces the correlation between spikes and the LFP in mouse primary visual cortex (V1), both under anesthesia (Nauhaus et al., 2009) and in wakefulness (Ray and Maunsell, 2011). Synaptic mechanisms underlying these changes in the relationship between the LFP and single neurons remain unclear.

Answering these questions has been hampered by two difficulties. First, previous studies of the LFP's relationship to excitation and inhibition were performed in vitro or under anesthesia.

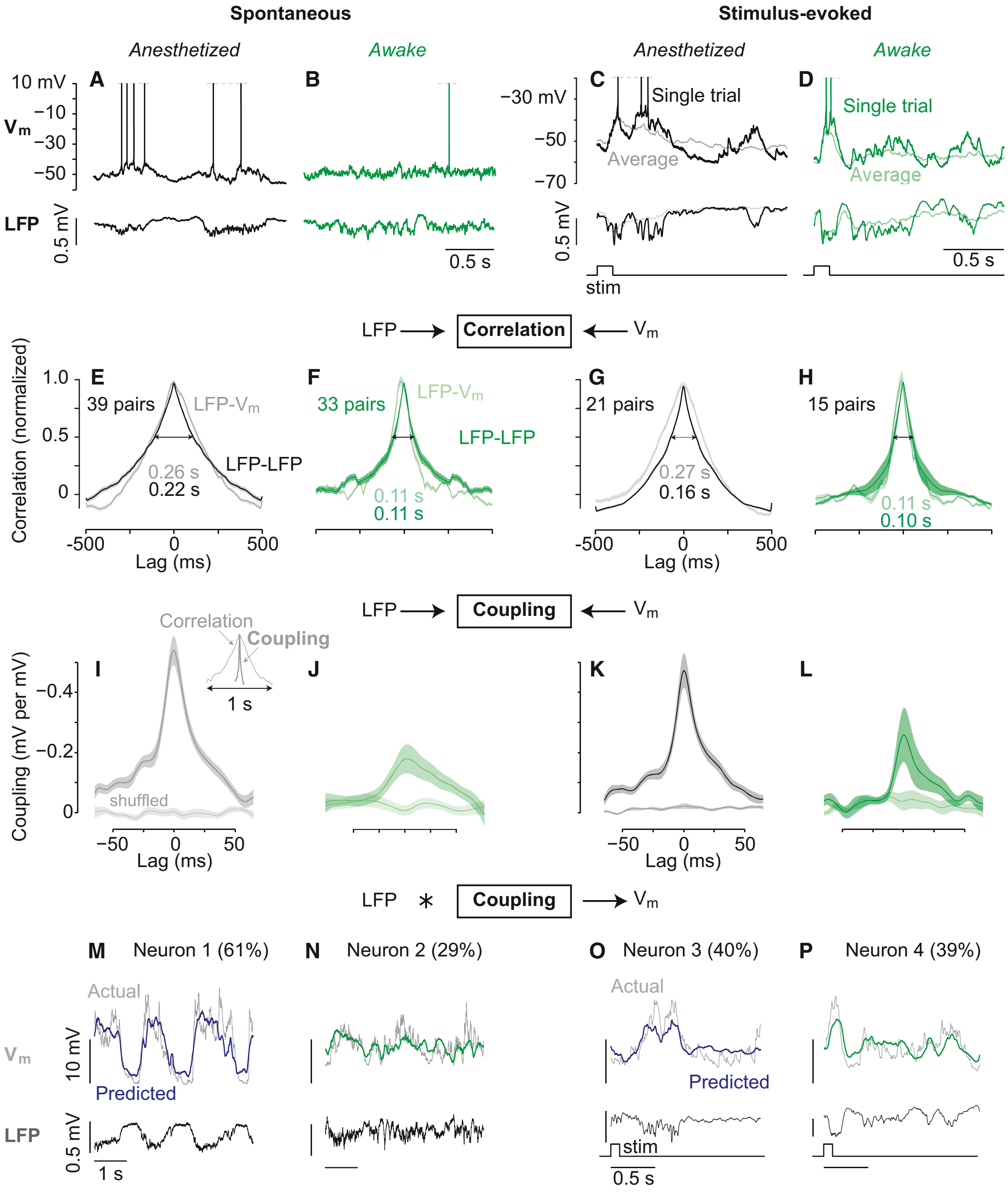

Figure 1. Fast Coupling between V_m and LFP in Cortical Area V1

(A) LFP and simultaneous whole-cell patch-clamp recording of V_m in L2/3 of anesthetized mouse V1 during spontaneous activity.
(B) As in (A), during wakefulness.
(C and D) Same as (A) and (B), during visual stimulation (bottom). Single-trial (darker) and average responses to ten trials (lighter). Spikes truncated at −20 mV. (A)–(D), four separate neurons.
(E and F) Normalized crosscorrelation of spontaneous LFP and V_m under anesthesia (E) and wakefulness (F). Light traces, mean across pairs ± SEM (shaded). Dark traces, autocorrelation of LFP. Values are full width at half maximum (arrows).
(G and H) Same as (E) and (F), during visual stimulation.
(I and J) Optimal coupling filters between LFP and V_m for spontaneous activity under anesthesia (I) or wakefulness (J). Shuffling trials destroys coupling filters. Inset: coupling filter at same timescale as correlation in (E).
(K and L) Same as (I) and (J), during visual stimulation.
(M and N) Coupling filters predict single-trial spontaneous V_m (top) from LFP (bottom) during anesthesia (M) or wakefulness (N). Crossvalidated coupling filters were convolved with LFP to predict simultaneous V_m (blue). Percentages indicate explained variance of predicted trace compared to actual trace (gray).
(O and P) Same as (M) and (N), during visual stimulation (bottom). (M)–(P), four separate neurons. See also Figures S1 and S2.

Anesthesia is not expected to alter the physics of extracellular current sources and sinks that generate the LFP; however, anesthesia does alter neuronal interactions at many timescales (Ecker et al., 2014). Second, the common method to relate LFP to single-neuron activity is to calculate crosscorrelations. These correlations are contaminated by the autocorrelations of the signals, which are dominated by shared slow fluctuations even during wakefulness (Okun et al., 2010; Poulet and Petersen, 2008). Even if the relationship between LFP and membrane potential (V_m) was instantaneous, their crosscorrelation would reflect the timescale of shared autocorrelations.

Here, we overcome these limitations using simultaneous patch-clamp and LFP recordings in the anesthetized and awake mouse visual cortex. We introduce a linear regularized method that separates the influence of slow autocorrelations from the fast (millisecond) coupling of LFP with intracellular activity. This method allows us to quantify how well the LFP predicts subthreshold excitatory and inhibitory activity of single cortical neurons.

RESULTS

We recorded the LFP in V1 and simultaneously measured intracellular activity in layer 2/3 with whole-cell patch-clamp recordings. We first made current-clamp recordings to investigate the relationship between LFP and V_m. In a second set of experiments, we recorded in voltage clamp while blocking intrinsic currents. We could thus assess the role of synaptic currents and the relationship between the LFP and synaptic excitation and inhibition. To study the effects of brain state and of sensory drive, we made our measurements in anesthetized and awake mice, and in the presence and absence of a brief visual stimulus. All procedures were made in accordance with the Animals (Scientific Procedures) Act 1986, UK.

Fast Coupling of LFP to Membrane Potential

The LFP bears close similarity to the V_m of nearby pyramidal neurons, but the relevant timescale of this relationship is obscured by shared slow fluctuations (Figures 1A–1D). This relationship is commonly characterized by computing crosscorrelations between the two signals (Figures 1E, 1F, and S1, available online). The temporal extent of crosscorrelations is dominated by slow fluctuations in the LFP. Indeed, under anesthesia, the timescale of LFP-V_m correlation was as broad as the LFP's autocorrelation (Figure 1E). During wakefulness, LFP-V_m correlation was smaller (Figure S1) and briefer but was again as broad as the LFP's autocorrelation (Figure 1F).

The presence of a visual stimulus hardly changed the timescale of LFP-V_m correlation. Stimulus-evoked correlations had similar timescale as spontaneous correlations, for anesthetized (Figure 1G) or awake (Figure 1H) visual responses, and for both signal and noise correlations (Figure S1). Therefore, regardless of brain state or sensory condition, correlations between LFP and V_m are dominated by slow fluctuations shared between population activity and cellular activity.

To understand the fast relationship between LFP and single neurons, we implemented a linear model that infers the optimal function needed to reproduce intracellular activity from the simultaneously recorded LFP. We used crossvalidated regularized linear regression to obtain these optimal functions (coupling filters; Mackay, 1992; Sahani and Linden, 2002). This method avoids overfitting while robustly estimating coupling filters independent from the influence of slow autocorrelations (see Supplemental Experimental Procedures). Convolving the LFP with the coupling filter yields a prediction of the intracellular signals. The accuracy of prediction enables assessment of the strength of coupling. We quantified this as the percentage of variance in the intracellular signal explained by the LFP. Explained variance ranges from 0 (for a constant model that merely predicts the mean) to 1 (perfect prediction).

This procedure yielded coupling filters whose bandwidth was optimized via crossvalidation, and that were an order of magnitude briefer than crosscorrelations, for both spontaneous (Figures 1I and 1J) and evoked activity (Figures 1K and 1L). Shuffling LFP-V_m pairing across trials (destroying simultaneity) produced flat coupling filters. This demonstrates that fast, millisecond coupling between LFP and V_m occurs uniquely within individual trials and does not simply reflect average stimulus onsets across trials. In agreement with correlations (Figure S1), spontaneous and evoked LFP-V_m coupling was significantly larger during anesthesia (filter area was as follows: 25 ± 0.2 and 22 ± 0.4, n = 39 and 21; mean ± SEM) than during wakefulness (11 ± 0.5 and 10 ± 0.8, n = 33 and 15; $p < 0.01$; Kruskal-Wallis ANOVA). Similar effects were observed with spike-triggered LFP (Figure S2).

Changes in the magnitude of coupling filters could reflect changes in the amplitude of the underlying signals, or changes in the ability of one signal to predict the other. To distinguish these possibilities, we convolved the LFP traces with the coupling filters to generate predicted V_m traces. As illustrated for four example neurons (Figures 1M–1P), these LFP predictions captured a substantial portion of simultaneously recorded V_m variance. Across all conditions, LFP predicted V_m better during anesthesia than during wakefulness (results for spontaneous activity are as follows: 38.0% ± 2.4% versus 26.7% ± 4.2% explained variance; results for evoked activity are as follows: 35.7% ± 3.2% versus 22.3% ± 3.7%, mean ± SEM). Simulations showed that regularized coupling filters significantly outperformed both unregularized linear regression and filters obtained directly from crosscorrelograms (Figure S2). This method accurately recreated subthreshold single-neuron activity from the surrounding population activity, and the changes in coupling reflect how accurately the cortical LFP predicts intracellular activity.

Fast Coupling between LFP and Synaptic Currents

We next investigated the relationship between the LFP and synaptic currents (Figure 2). We blocked spikes and most other intrinsic currents in the recorded neuron (see Supplemental Experimental Procedures). We held membrane potential near −80 mV to measure excitatory postsynaptic currents (EPSCs; Figure 2A) or near +20 mV to measure inhibitory postsynaptic currents (IPSCs; Figure 2B). We then used the same methods as before to obtain the coupling between those synaptic currents and the LFP (Figures 2C–2F).

LFP coupling to synaptic currents showed consistent differences in timing, with excitation reliably preceding inhibition (Figures 2C–2F). During anesthesia, LFP-EPSC coupling preceded

Cell Press

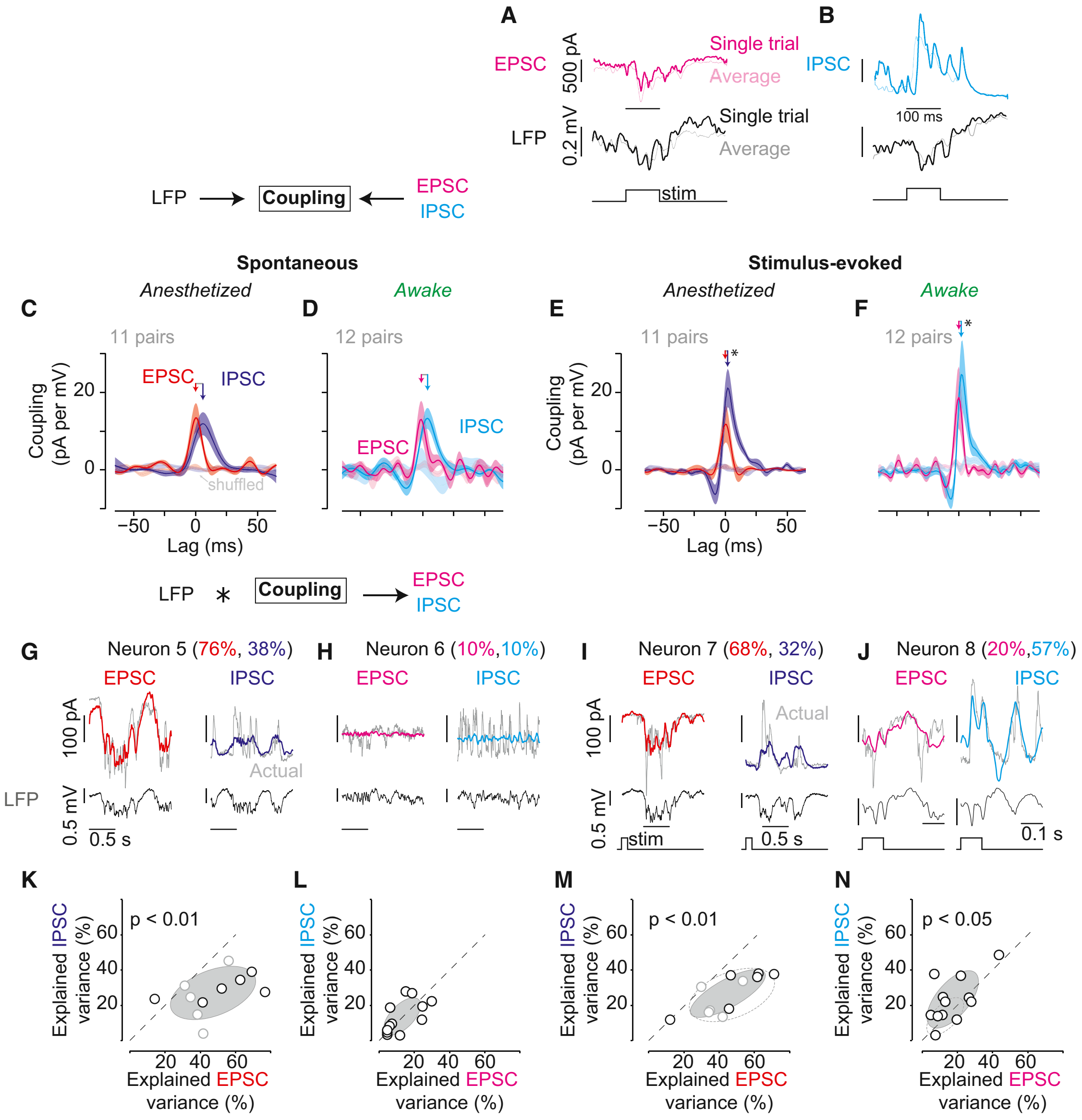

Figure 2. Fast Coupling between LFP and Synaptic Currents

(A and B) EPSCs (magenta) or IPSCs (cyan) recorded from the same neuron simultaneously with LFP (black, bottom). Single-trial (dark) and average responses (light) during awake visual stimulation (flashed bar, bottom). EPSCs recorded at −80 mV and IPSCs at +20 mV, respectively.

(C and D) Population coupling filters between LFP and PSCs during anesthetized (left) and awake (right) spontaneous activity. Filters from shuffled trials are flat (lighter colors). Mean ± SEM (shaded) shown for all.

(E and F) Population coupling filters between LFP and PSCs during stimulus-evoked activity. Same neurons as (C) and (D). Significantly faster excitatory-inhibitory lag during stimulus-evoked activity versus spontaneous (paired Wilcoxon signed-rank, $p < 0.01$ for both).

(G and H) Coupling filters convolved with LFP (bottom) predict single-trial spontaneous synaptic currents (EPSC or IPSC, top) during anesthesia (G) and wakefulness (H). Same scales throughout. Percentages indicate explained variance of predicted traces (colored) compared to the actual trace (gray).

(I and J) Same as (G) and (H), during visual stimulation. (G)–(J), four separate neurons.

(K and L) Prediction quality (percent of explained variance) of EPSCs and IPSCs from spontaneous LFP during anesthesia (K) and wakefulness (L). Shaded regions, 2D Gaussian fit ± 1 σ. EPSCs significantly better predicted than IPSCs under anesthesia (repeated-measures ANOVA, $p < 0.01$). In (K), lighter and darker points indicate urethane and isoflurane, respectively.

(M and N) Prediction quality of EPSCs and IPSCs from stimulus-evoked LFP. During anesthesia (M), LFP predicts EPSCs significantly better than IPSCs (paired Wilcoxon signed-rank, $p < 0.01$). During wakefulness (N) LFP predicts IPSCs better than EPSCs (paired Wilcoxon signed-rank, $p < 0.05$). Total evoked currents are significantly more predictable than spontaneous currents during wakefulness (repeated-measures ANOVA, $p < 0.01$). Dashed ellipse shows fits from (K) and (L). In (M), lighter and darker points indicate anesthetic as in (K). See also Figures S3–S5.

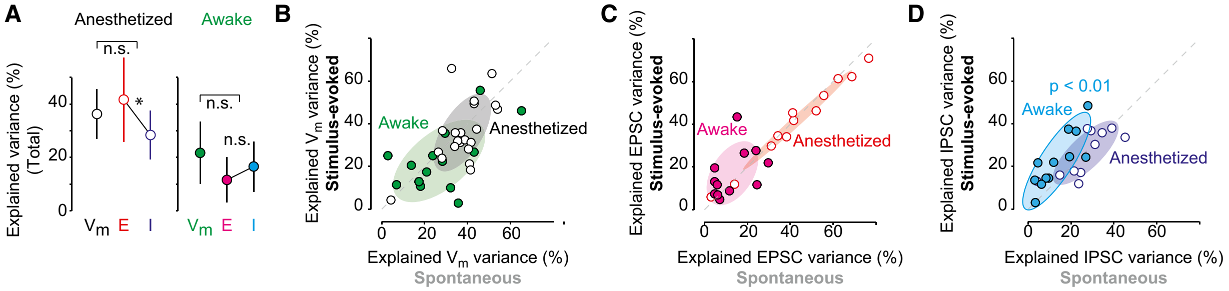

Figure 3. Wakefulness and Visual Stimuli Enhance Coupling of LFP to Inhibition

(A) LFP prediction of V_m, EPSCs, and IPSCs during anesthesia (left, n = 40, 22, 22) and wakefulness (right, n = 30, 24, 24). During anesthesia, EPSCs are significantly more predictable than IPSCs ($p = 0.01$; repeated-measures ANOVA; n = 22 for both). No significant difference in total LFP predictions of V_m versus PSCs across states. Median of all spontaneous and stimulus-evoked predictions calculated within neuron then averaged across populations (median ± MAD [median absolute deviation]).

(B) Prediction quality (percent of explained variance) of spontaneous versus stimulus-evoked V_m during anesthesia (n = 21) and wakefulness (n = 15). Shaded regions, 2D Gaussian fit ± 1 σ throughout figure. No significant effect of stimulus within groups ($p = 0.2$; $p = 0.4$).

(C) Prediction quality of spontaneous versus stimulus-evoked EPSCs during anesthesia (red) and wakefulness (magenta). No significant effect of stimulus within groups ($p = 0.2$; $p = 0.09$).

(D) Stimulus-evoked IPSCs are significantly more predictable from LFP than spontaneous IPSCs during wakefulness ($p < 0.01$; paired Wilcoxon signed-rank). No significant effect during anesthesia ($p = 0.8$). See also Figures S3–S5.

LFP-IPSC coupling by 4.5 ± 0.7 ms (Figure 2C; n = 11 pairs). Visual stimulation significantly shortened this lag to 1.0 ± 0.7 ms (Figure 2E; $p < 0.01$; paired Wilcoxon signed-rank). These delays between excitation and inhibition are consistent with values measured in previous anesthetized work (Atallah and Scanziani, 2009; Hasenstaub et al., 2005; Okun and Lampl, 2008; Poo and Isaacson, 2009).

LFP coupling to inhibition lagged coupling to excitation during wakefulness; this lag was again significantly shortened by sensory stimulation (from 3.7 ± 1.0 ms to 0.4 ± 1.1 ms, n = 12 pairs; $p < 0.01$; paired Wilcoxon signed-rank). These millisecond timing differences in LFP coupling to synaptic activity could not have been observed from crosscorrelations (Figure S1).

Brain state strongly influenced predictions of synaptic currents from the LFP. Under anesthesia, EPSCs were significantly better predicted than IPSCs across conditions (Figure 2K; results for spontaneous activity are as follows: 43.3% ± 6.2% versus 26.8% ± 3.4%; Figure 2M; results for evoked activity are as follows: 41.5% ± 5.7% versus 26.2% ± 3.3%, n = 11 pairs; $p < 0.01$ for both; paired Wilcoxon signed-rank). During wakefulness, instead, the LFP predicted spontaneous EPSCs and IPSCs equally (Figure 2L; 13.1% ± 2.6% and 17.0% ± 3.3%, n = 12 pairs). Moreover, the awake LFP predicted stimulus-evoked IPSCs significantly better than EPSCs (Figure 2N; 22.7% ± 3.7% and 13.4% ± 2.6%; $p < 0.05$; paired Wilcoxon signed-rank). These results show that the anesthetized LFP overwhelmingly reports activity of excitatory circuits; in contrast, the awake LFP reports spontaneous activity of excitatory and inhibitory circuits equally and predicts stimulus-evoked inhibition better than excitation.

During wakefulness, the strength of LFP coupling to the total synaptic current reflected stimulus conditions: without a stimulus, the LFP explained 13.2% ± 1.8% of the variance of postsynaptic currents (Figure 2L; EPSCs and IPSCs combined, n = 24), but with a stimulus it explained 19.9% ± 2.5% (Figure 2N; $p < 0.01$; repeated-measures ANOVA). This effect of stimuli was absent during anesthesia (35.4% ± 4.0% versus 34.2% ± 3.7%, n = 22).

Wakefulness and Stimuli Enhance Coupling of LFP to Inhibition

We next assessed whether the LFP more accurately predicts V_m or synaptic currents. We compared our two recording conditions: when measuring V_m, and when measuring only EPSCs and IPSCs, while pooling across spontaneous and sensory conditions (Figure 3A). During anesthesia, predictions of V_m (n = 42) and EPSCs (n = 22) were similarly accurate, while IPSCs (n = 22) were significantly less predictable ($p = 0.01$; repeated-measures ANOVA). During wakefulness, instead, the LFP provided equally faithful predictions of V_m (n = 30), EPSCs (n = 24), and IPSCs (n = 24). In both anesthesia and wakefulness, there was no significant difference between the predictability of V_m and of total synaptic currents. In additional experiments where we recorded both V_m and EPSCs within the same neurons and without blocking intrinsic conductances (n = 5), we found that predictions of V_m and EPSCs were nearly identical with one another ($r = 0.95$; $p < 0.01$; data not shown). Taken together, these results indicate that synaptic currents provide the main contribution for prediction of cellular activity from the LFP.

During wakefulness, LFP coupling to synaptic currents was further enhanced by sensory stimulation (Figures 3B–3D). Visually evoked EPSCs tended to be more predictable than spontaneous ones (Figure 3C; 17.0% ± 3.3% versus 13.1% ± 2.6%, n = 12; $p = 0.09$; paired Wilcoxon signed-rank), but stimulation did not improve predictions of EPSCs during anesthesia (Figure 3C), and did not improve LFP predictions of V_m (Figure 3B) in either brain state.

The effect of visual stimulation was most pronounced for LFP coupling to IPSCs during wakefulness (Figure 3D). Visual stimulation significantly improved LFP-IPSC predictions (22.7% ± 3.7% versus 13.4% ± 2.6%, n = 12; $p < 0.01$; paired Wilcoxon signed-rank). Again, stimuli did not cause these effects on IPSCs

during anesthesia. Taken together, these results show that the awake LFP reliably reflects the influence of both excitation and inhibition and accurately reports changes in synaptic input driven by sensory stimulation.

Could distance between the intracellular and LFP electrodes account for differences across cells? We did not observe a significant correlation of LFP prediction quality across the distances we sampled (Supplemental Experimental Procedures; Figures S3 and S4; $p > 0.05$), except during anesthesia, and only in specific frequency bands. The heterogeneity in LFP coupling may arise from other factors (Okun et al., 2015).

Finally, we asked whether differences in LFP coupling could be observed during trial-by-trial fluctuations of cortical state. To this end, we used the LFP to classify cortical state on individual trials (Figure S5). This analysis indicated that spontaneous fluctuations in cortical state caused significant changes in the strength of LFP coupling to subthreshold activity.

DISCUSSION

We have shown that the LFP can be used to predict the subthreshold synaptic input to individual neurons in visual cortex. In intact and awake conditions, the LFP contains substantial predictive power for millisecond changes in membrane potential, synaptic excitation, and synaptic inhibition. These findings provide constraints for computational simulations of neocortex (Izhikevich and Edelman, 2008; Markram et al., 2015) and lend mechanistic insight for macroscopic electrical signals (such as EEG and ECoG) recorded in the awake human cortex.

A first consequence of our findings concerns the relationship of the LFP to synaptic and intrinsic currents in single neurons. From the extracellular point of view, both sets of currents contribute directly to the LFP (Buzsáki et al., 1988, 2012; Einevoll et al., 2013; Jefferys and Haas, 1982; Lindén et al., 2011; Reimann et al., 2013). We assessed the ability of the LFP to predict activity in single neurons when these currents were intact, or largely suppressed. If nonsynaptic currents play a major role, then the LFP's ability to predict membrane potential (intrinsic conductances intact) should be superior to its ability to predict synaptic currents alone. Instead, under our experimental conditions, we found that the LFP predicted relatively well-isolated EPSCs or IPSCs as well as it predicted membrane potential, indicating that synaptic currents play a central role in the functional relationship between the LFP and single-neuron activity.

A second consequence of our findings concerns the relative contributions of synaptic excitation and inhibition to the LFP. Confirming previous views, we found that the LFP was strongly coupled to excitation under anesthesia. However, during wakefulness the LFP predicted synaptic inhibition and excitation more equally. These findings suggest more effective synchronization of pyramidal neurons by common inhibitory inputs during wakefulness, consistent with an enhanced role for inhibition in awake conditions (Haider et al., 2013; Rudolph et al., 2007).

A third consequence of our findings concerns the effect of sensory stimuli on the relationship between the LFP and synaptic currents. In V1, visual stimulation reduces the correlation between spike trains and the nearby LFP (Nauhaus et al., 2009; Ray and Maunsell, 2011). Our results suggest a synaptic basis for these effects during wakefulness: the awake LFP predicted IPSCs better during visual stimulation than during spontaneous activity (Figure 3D), and during visual stimulation, it predicted IPSCs better than EPSCs (Figure 2N). These results indicate that during wakefulness, sensory stimulation strengthens the coupling between LFP and inhibitory activity, relative to excitatory activity. Prominent inhibition improves awake sensory processing (Haider et al., 2013) and may also decorrelate evoked spikes from the overall population response visible in the LFP.

One of the advantages of our method of estimating coupling filters is that it revealed temporal relationships at a fast timescale. As expected from the effects of membrane capacitance, the time course of LFP coupling to synaptic currents was briefer than coupling to V_m. The coupling of LFP to synaptic excitation, moreover, preceded the coupling to synaptic inhibition by a few milliseconds, a lag that was reduced upon visual stimulation. These observations in the intact and awake cortex extend previous measurements made during oscillations in vitro (Mann et al., 2005; Oren et al., 2006) and under anesthesia in vivo (Atallah and Scanziani, 2009; Hasenstaub et al., 2005; Okun and Lampl, 2008; Penttonen et al., 1998; Poo and Isaacson, 2009). Synaptic activity recorded at the soma likely reflects synchronized firing in presynaptic excitatory and inhibitory populations. In fact, all coupling filters peaked at positive lags, indicating that the LFP slightly precedes the currents visible at the soma. The observed lag of somatic inhibition versus excitation may stem in part from the delay of fast-spiking interneuron firing relative to excitatory neurons (Hasenstaub et al., 2005; Luczak et al., 2007; Salkoff et al., 2015). Some excitatory inputs to layer 2/3 also originate from thalamic afferents (Kloc and Maffei, 2014), providing a further temporal advantage to excitation during visual stimulation.

Because our method estimates coupling filters in the temporal domain, it does not require one to define frequency bands whose relevance varies across single trials, subjects, brain states, and sensory conditions (Jia et al., 2013; Kayser et al., 2003; Ray and Maunsell, 2010). However, our method does implicitly emphasize frequencies <100 Hz (determined by the hyperparameters that minimized prediction error). Further studies employing alternative predictive methods (Park and Pillow, 2011; Rasch et al., 2009) could further explore LFP coupling to synaptic activity, including higher frequencies (>100 Hz) where the LFP may reflect spike activity (Teleńczuk et al., 2015).

How much of the intracellular activity in single cortical neurons can be predicted from the LFP? Under anesthesia, the LFP can predict 20%–60% of the variance in V_m and EPSCs and 20%–40% of the variance in IPSCs. During wakefulness, these numbers are lower, especially for V_m and EPSCs. Yet these numbers indicate a surprising degree of predictability. Explained variance varied approximately as the square of correlation, so an apparently small amount of explained variance such as 0.25 corresponds in fact to a correlation of 0.5. This appears consistent with previous reports of V_m-LFP correlations (ranging from 0.3 to 0.6) in awake cortex sampling similar distances as our study (Okun et al., 2010; Poulet and Petersen, 2008). It would be surprising if single-neuron activity was captured perfectly by a global signal such as the LFP; this would imply that large populations of neurons behave identically.

Our study focused on predicting single-neuron activity from the LFP, not on the genesis of the LFP signal itself, and comes with a number of limitations. First, as with all somatic recordings, our results are biased toward synaptic activity generated in proximal portions of the neuron (Williams and Mitchell, 2008). The thin distal dendrites of pyramidal cells contain many active conductances that are difficult to detect using somatic recordings and are not well-controlled by somatic voltage clamp. These dendrites may contribute substantially to the LFP; it will be important in future investigations to understand LFP coupling to both somatic and dendritic signals. Second, we concentrated on intracellular signals measured in pyramidal neurons in L2/3 of mouse V1; it will be interesting to extend this approach to other cell types (e.g., interneurons), other layers, and other neural circuits with different connectivity and synaptic dynamics. Third, we concentrated on spontaneous activity and on activity elicited during passive presentation of a brief stimulus in mouse V1. It remains to be seen how the LFP is coupled to synaptic activity in different conditions and brain states, for instance, during behavioral tasks.

EXPERIMENTAL PROCEDURES

Please see Supplemental Experimental Procedures for the full Experimental Procedures.

SUPPLEMENTAL INFORMATION

Supplemental Information includes Supplemental Experimental Procedures and five figures and can be found with this article online at http://dx.doi.org/10.1016/j.neuron.2016.02.034.

AUTHOR CONTRIBUTIONS

B.H. performed experiments; D.P.A.S. developed filter methods; B.H., D.P.A.S., and M.C. analyzed data; and all authors discussed results and interpretation and wrote the manuscript.

ACKNOWLEDGMENTS

We thank Chris Burgess for help during experiments, Michael Okun for discussions, and Mehmet Fisek for comments on the manuscript. This work was funded by the National Science Foundation (NSF IRFP 0965110), the European Research Council, and the Wellcome Trust. M.C. holds the GlaxoSmithKline/Fight for Sight Chair in Visual Neuroscience.

Received: April 23, 2015
Revised: October 23, 2015
Accepted: February 16, 2016
Published: March 24, 2016

REFERENCES

Atallah, B.V., and Scanziani, M. (2009). Instantaneous modulation of gamma oscillation frequency by balancing excitation with inhibition. Neuron *62*, 566–577.

Bartos, M., Vida, I., and Jonas, P. (2007). Synaptic mechanisms of synchronized gamma oscillations in inhibitory interneuron networks. Nat. Rev. Neurosci. *8*, 45–56.

Bazelot, M., Dinocourt, C., Cohen, I., and Miles, R. (2010). Unitary inhibitory field potentials in the CA3 region of rat hippocampus. J. Physiol. *588*, 2077–2090.

Braitenberg, V., and Schuz, A. (1991). Anatomy of the cortex (Springer-Verlag).

Buzsáki, G., Bickford, R.G., Ponomareff, G., Thal, L.J., Mandel, R., and Gage, F.H. (1988). Nucleus basalis and thalamic control of neocortical activity in the freely moving rat. J. Neurosci. *8*, 4007–4026.

Buzsáki, G., Anastassiou, C.A., and Koch, C. (2012). The origin of extracellular fields and currents–EEG, ECoG, LFP and spikes. Nat. Rev. Neurosci. *13*, 407–420.

Destexhe, A., Contreras, D., and Steriade, M. (1999). Spatiotemporal analysis of local field potentials and unit discharges in cat cerebral cortex during natural wake and sleep states. J. Neurosci. *19*, 4595–4608.

Ecker, A.S., Berens, P., Cotton, R.J., Subramaniyan, M., Denfield, G.H., Cadwell, C.R., Smirnakis, S.M., Bethge, M., and Tolias, A.S. (2014). State dependence of noise correlations in macaque primary visual cortex. Neuron *82*, 235–248.

Einevoll, G.T., Kayser, C., Logothetis, N.K., and Panzeri, S. (2013). Modelling and analysis of local field potentials for studying the function of cortical circuits. Nat. Rev. Neurosci. *14*, 770–785.

Glickfeld, L.L., Roberts, J.D., Somogyi, P., and Scanziani, M. (2009). Interneurons hyperpolarize pyramidal cells along their entire somatodendritic axis. Nat. Neurosci. *12*, 21–23.

Haider, B., Häusser, M., and Carandini, M. (2013). Inhibition dominates sensory responses in the awake cortex. Nature *493*, 97–100.

Hasenstaub, A., Shu, Y., Haider, B., Kraushaar, U., Duque, A., and McCormick, D.A. (2005). Inhibitory postsynaptic potentials carry synchronized frequency information in active cortical networks. Neuron *47*, 423–435.

Hubbard, J.I., Llinas, R.R., and Quastel, D.M.J. (1969). Electrophysiological analysis of synaptic transmission (Williams & Wilkins Co.).

Izhikevich, E.M., and Edelman, G.M. (2008). Large-scale model of mammalian thalamocortical systems. Proc. Natl. Acad. Sci. USA *105*, 3593–3598.

Jefferys, J.G., and Haas, H.L. (1982). Synchronized bursting of CA1 hippocampal pyramidal cells in the absence of synaptic transmission. Nature *300*, 448–450.

Jia, X., Tanabe, S., and Kohn, A. (2013). γ and the coordination of spiking activity in early visual cortex. Neuron *77*, 762–774.

Katzner, S., Nauhaus, I., Benucci, A., Bonin, V., Ringach, D.L., and Carandini, M. (2009). Local origin of field potentials in visual cortex. Neuron *61*, 35–41.

Kayser, C., Salazar, R.F., and Konig, P. (2003). Responses to natural scenes in cat V1. J. Neurophysiol. *90*, 1910–1920.

Kloc, M., and Maffei, A. (2014). Target-specific properties of thalamocortical synapses onto layer 4 of mouse primary visual cortex. J. Neurosci. *34*, 15455–15465.

Lindén, H., Tetzlaff, T., Potjans, T.C., Pettersen, K.H., Grün, S., Diesmann, M., and Einevoll, G.T. (2011). Modeling the spatial reach of the LFP. Neuron *72*, 859–872.

Logothetis, N.K., Pauls, J., Augath, M., Trinath, T., and Oeltermann, A. (2001). Neurophysiological investigation of the basis of the fMRI signal. Nature *412*, 150–157.

Luczak, A., Barthó, P., Marguet, S.L., Buzsáki, G., and Harris, K.D. (2007). Sequential structure of neocortical spontaneous activity in vivo. Proc. Natl. Acad. Sci. USA *104*, 347–352.

Mackay, D.J.C. (1992). Bayesian interpolation. Neural Comput. *4*, 415–447.

Mann, E.O., Suckling, J.M., Hajos, N., Greenfield, S.A., and Paulsen, O. (2005). Perisomatic feedback inhibition underlies cholinergically induced fast network oscillations in the rat hippocampus in vitro. Neuron *45*, 105–117.

Markram, H., Muller, E., Ramaswamy, S., Reimann, M.W., Abdellah, M., Sanchez, C.A., Ailamaki, A., Alonso-Nanclares, L., Antille, N., Arsever, S., et al. (2015). Reconstruction and simulation of neocortical microcircuitry. Cell *163*, 456–492.

Mitzdorf, U., and Singer, W. (1978). Prominent excitatory pathways in the cat visual cortex (A 17 and A 18): a current source density analysis of electrically evoked potentials. Exp. Brain Res. *33*, 371–394.

Nauhaus, I., Busse, L., Carandini, M., and Ringach, D.L. (2009). Stimulus contrast modulates functional connectivity in visual cortex. Nat. Neurosci. *12*, 70–76.

Okun, M., and Lampl, I. (2008). Instantaneous correlation of excitation and inhibition during ongoing and sensory-evoked activities. Nat. Neurosci. *11*, 535–537.

Okun, M., Naim, A., and Lampl, I. (2010). The subthreshold relation between cortical local field potential and neuronal firing unveiled by intracellular recordings in awake rats. J. Neurosci. *30*, 4440–4448.

Okun, M., Steinmetz, N.A., Cossell, L., Iacaruso, M.F., Ko, H., Barthó, P., Moore, T., Hofer, S.B., Mrsic-Flogel, T.D., Carandini, M., and Harris, K.D. (2015). Diverse coupling of neurons to populations in sensory cortex. Nature *521*, 511–515.

Oren, I., Mann, E.O., Paulsen, O., and Hájos, N. (2006). Synaptic currents in anatomically identified CA3 neurons during hippocampal gamma oscillations in vitro. J. Neurosci. *26*, 9923–9934.

Park, M., and Pillow, J.W. (2011). Receptive field inference with localized priors. PLoS Comput. Biol. *7*, e1002219.

Penttonen, M., Kamondi, A., Acsády, L., and Buzsáki, G. (1998). Gamma frequency oscillation in the hippocampus of the rat: intracellular analysis in vivo. Eur. J. Neurosci. *10*, 718–728.

Poo, C., and Isaacson, J.S. (2009). Odor representations in olfactory cortex: "sparse" coding, global inhibition, and oscillations. Neuron *62*, 850–861.

Poulet, J.F., and Petersen, C.C. (2008). Internal brain state regulates membrane potential synchrony in barrel cortex of behaving mice. Nature *454*, 881–885.

Rasch, M., Logothetis, N.K., and Kreiman, G. (2009). From neurons to circuits: linear estimation of local field potentials. J. Neurosci. *29*, 13785–13796.

Ray, S., and Maunsell, J.H. (2010). Differences in gamma frequencies across visual cortex restrict their possible use in computation. Neuron *67*, 885–896.

Ray, S., and Maunsell, J.H. (2011). Network rhythms influence the relationship between spike-triggered local field potential and functional connectivity. J. Neurosci. *31*, 12674–12682.

Reimann, M.W., Anastassiou, C.A., Perin, R., Hill, S.L., Markram, H., and Koch, C. (2013). A biophysically detailed model of neocortical local field potentials predicts the critical role of active membrane currents. Neuron *79*, 375–390.

Rudolph, M., Pospischil, M., Timofeev, I., and Destexhe, A. (2007). Inhibition determines membrane potential dynamics and controls action potential generation in awake and sleeping cat cortex. J. Neurosci. *27*, 5280–5290.

Sahani, M., and Linden, J.F. (2002). Evidence optimization techniques for estimating stimulus-response functions. S. Becker, S. Thrun, and K. Obermayer, eds. Advances in Neural Information Processing Systems (NIPS) 12, 301–308.

Salkoff, D.B., Zagha, E., Yüzgeç, Ö., and McCormick, D.A. (2015). Synaptic mechanisms of tight spike synchrony at gamma frequency in cerebral cortex. J. Neurosci. *35*, 10236–10251.

Teleńczuk, B., Baker, S.N., Kempter, R., and Curio, G. (2015). Correlates of a single cortical action potential in the epidural EEG. Neuroimage *109*, 357–367.

Trevelyan, A.J. (2009). The direct relationship between inhibitory currents and local field potentials. J. Neurosci. *29*, 15299–15307.

Williams, S.R., and Mitchell, S.J. (2008). Direct measurement of somatic voltage clamp errors in central neurons. Nat. Neurosci. *11*, 790–798.

Neuron, Volume *90*

Supplemental Information

Millisecond Coupling of Local Field Potentials to Synaptic Currents in the Awake Visual Cortex

Bilal Haider, David P.A. Schulz, Michael Häusser, and Matteo Carandini

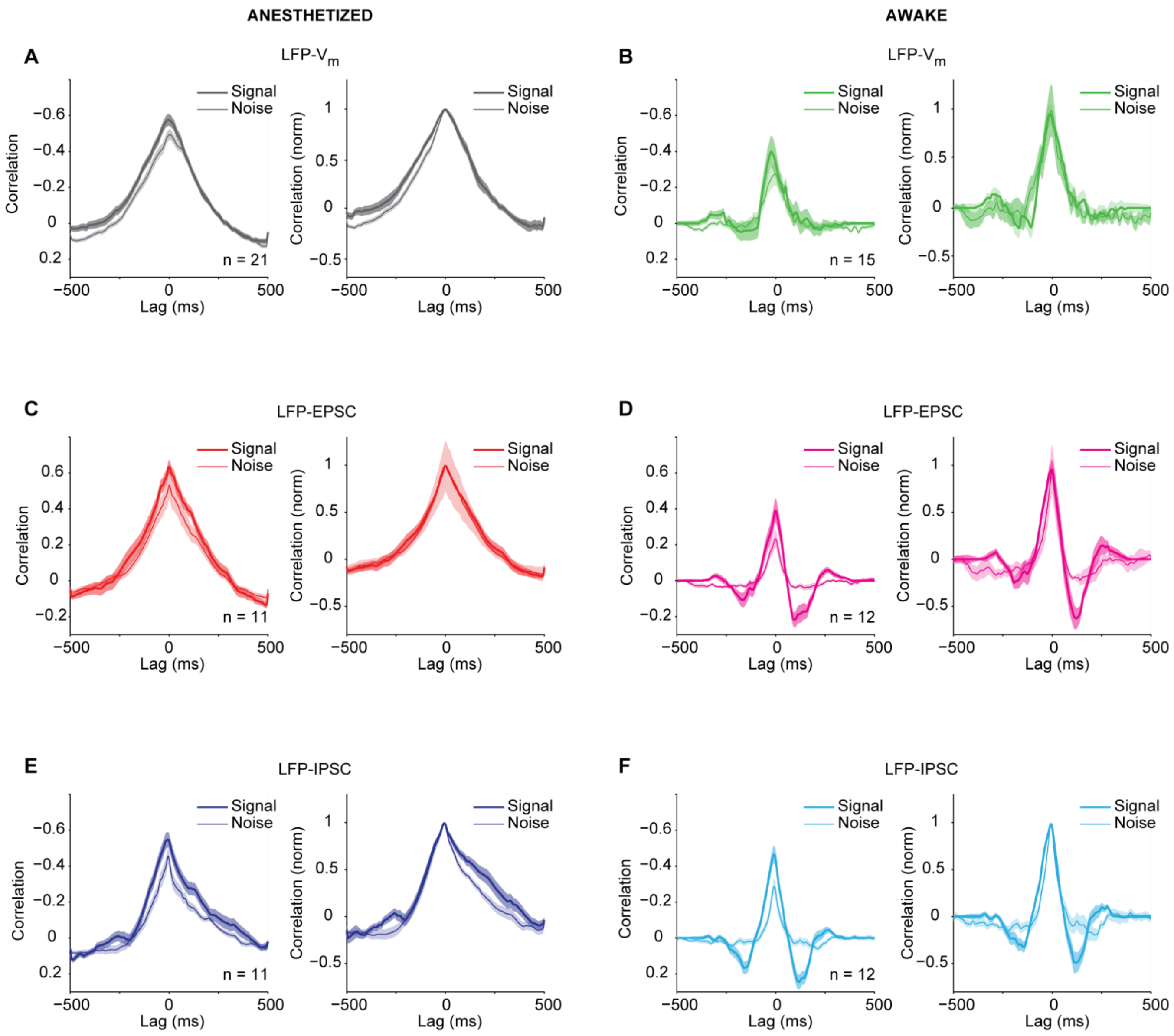

FIGURE S1 (related to Figure 1). Time course of anesthetized and awake signal and noise correlations.

A. LFP-V_m signal (darker) and noise (lighter) correlations during anesthesia (left), and signal and noise correlations normalized to peak (right). Note broad timescales. Noise correlations calculated by subtracting average stimulus response from every individual trial. Traces show median ± SEM (shaded).

B. Same as in A, for awake LFP- V_m signal and noise correlations.

C. Same as in A, for anesthetized LFP-EPSC signal and noise correlations.

D. Same as in A, for awake LFP-EPSC signal and noise correlations.

E. Same as in A, for anesthetized LFP-IPSC signal and noise correlations.

F. Same as in A, for awake LFP-IPSC signal and noise correlations.

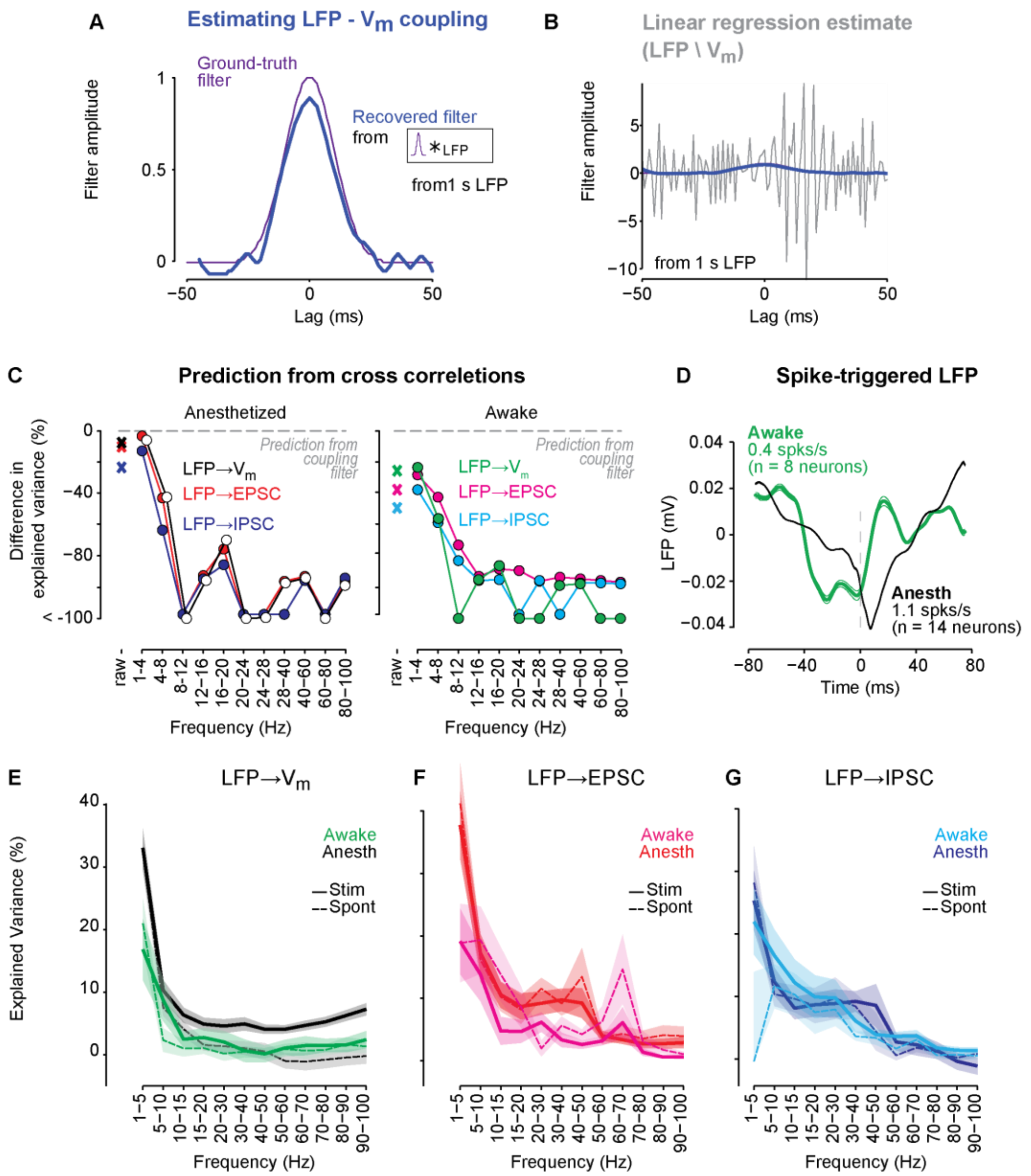

FIGURE S2 (related to Figures 1 and 2). Regularized filter estimation accurately recovers ground-truth coupling.

A. LFP traces (1 s snippets) were convolved with a ground-truth filter (Normalized Gaussian, violet) and contaminated with additive Gaussian noise to produce 'fake' V_m. Filter estimation procedure was then used to recover optimal coupling of LFP to 'fake' V_m (blue). Note similar shapes of ground-truth filter and recovered filter.

B. Filter estimated from unregularized linear regression (grey) on same data set fails to recover ground-truth coupling. The highly irregular shape of the estimated filter is due to overfitting high frequencies.

C. Predictions from cross correlations were substantially worse than predictions from coupling filters. Filters shaped exactly like cross correlations (for each individual paired recording, during visual stimulation) were used to predict intracellular traces from the LFP. We compared the variance explained by these predictions to variance explained by optimal coupling filters (% difference). Cross correlation predicts most poorly during wakefulness (Right: raw V_m (green) 26% worse, raw EPSCs (magenta) 38% worse, raw IPSCs (cyan) 50% worse). Cross correlations failed to predict information in higher frequencies (circles). Same population of neurons as in Figures 2 and 3.

D. Stimulus-evoked spike-triggered LFP (stLFP) during wakefulness (green) is narrower and smaller than during anesthesia (black). Mean ± SEM.

E-G. Explained variance as a function of frequency for LFP-Vm, LFP-EPSC, and LFP-IPSC coupling. Mean ± SEM.

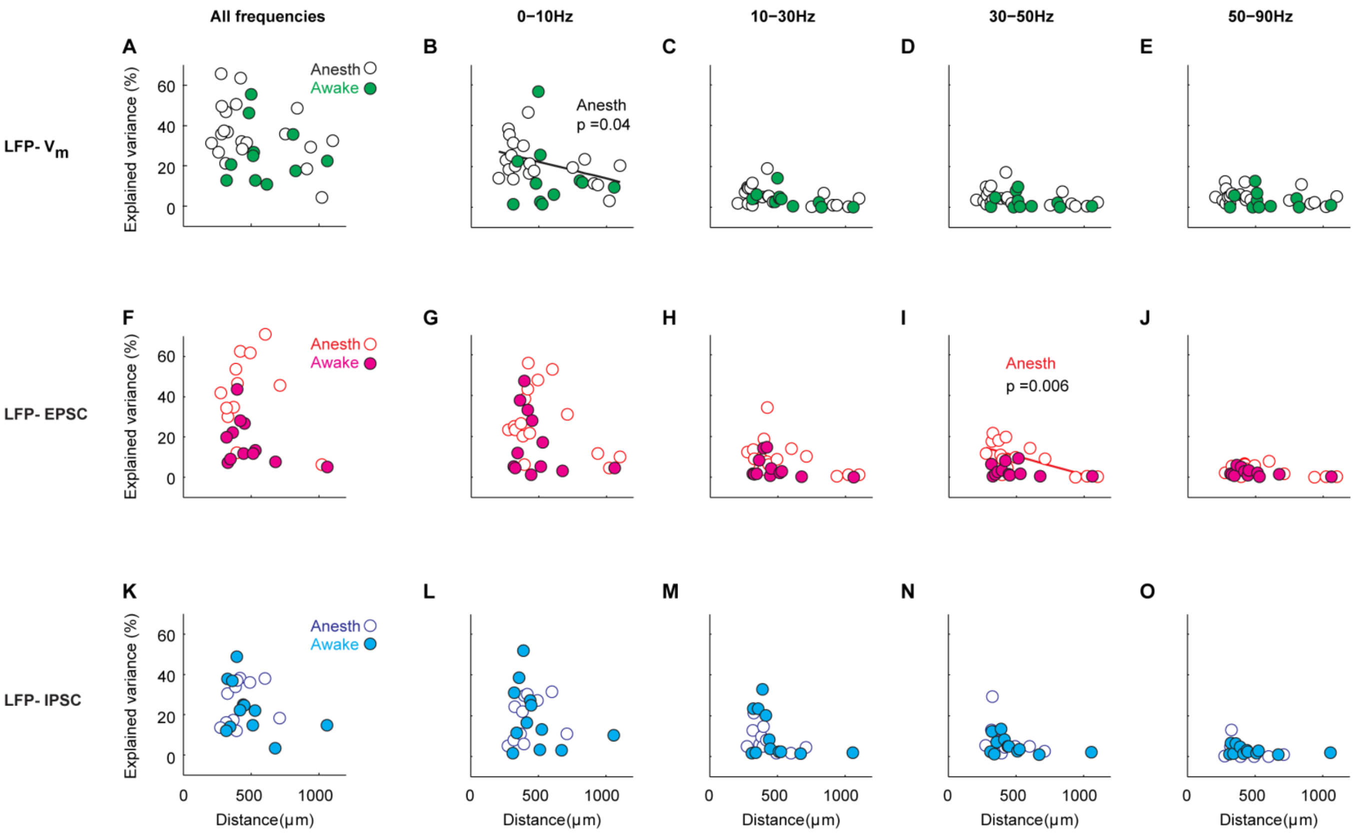

FIGURE S3 (Related to Figures 2 and 3). Explained variance as a function of frequency band and separation distance.

A-E. Stimulus-evoked LFP-V_m predictions during anesthetized and awake states. **A**, Explained variance of raw signals, as a function of separation between LFP and single neuron recording. **B**, Explained variance for signals filtered < 10Hz. Significant correlation with distance during anesthesia. **C**, Explained variance for signals filtered from 10 - 30Hz. **D**, Explained variance for signals filtered from 30 - 50Hz. **E**, Explained variance for signals filtered from 50 - 90Hz.

F-J. Stimulus-evoked LFP-EPSC predictions during anesthetized and awake states. **F**, Explained variance of raw signals, as a function of separation between LFP and single neuron recording **G**, Explained variance for signals filtered < 10Hz; **H**, Explained variance for signals filtered from 10 - 30Hz. **I.** Explained variance for signals filtered from 30 - 50Hz. Significant correlation during anesthesia. **J**, Explained variance for signals filtered from 50 - 90Hz.

K-O. Stimulus-evoked LFP-IPSC predictions during anesthetized and awake states. **K**, Explained variance of raw signals, as a function of separation between LFP and single neuron recording. **L**, Explained variance for signals filtered < 10Hz. **M**, Explained variance for signals filtered from 10 - 30Hz. **N,** Explained variance for signals filtered from 30 - 50Hz. **O**, Explained variance for signals filtered from 50 - 90Hz.

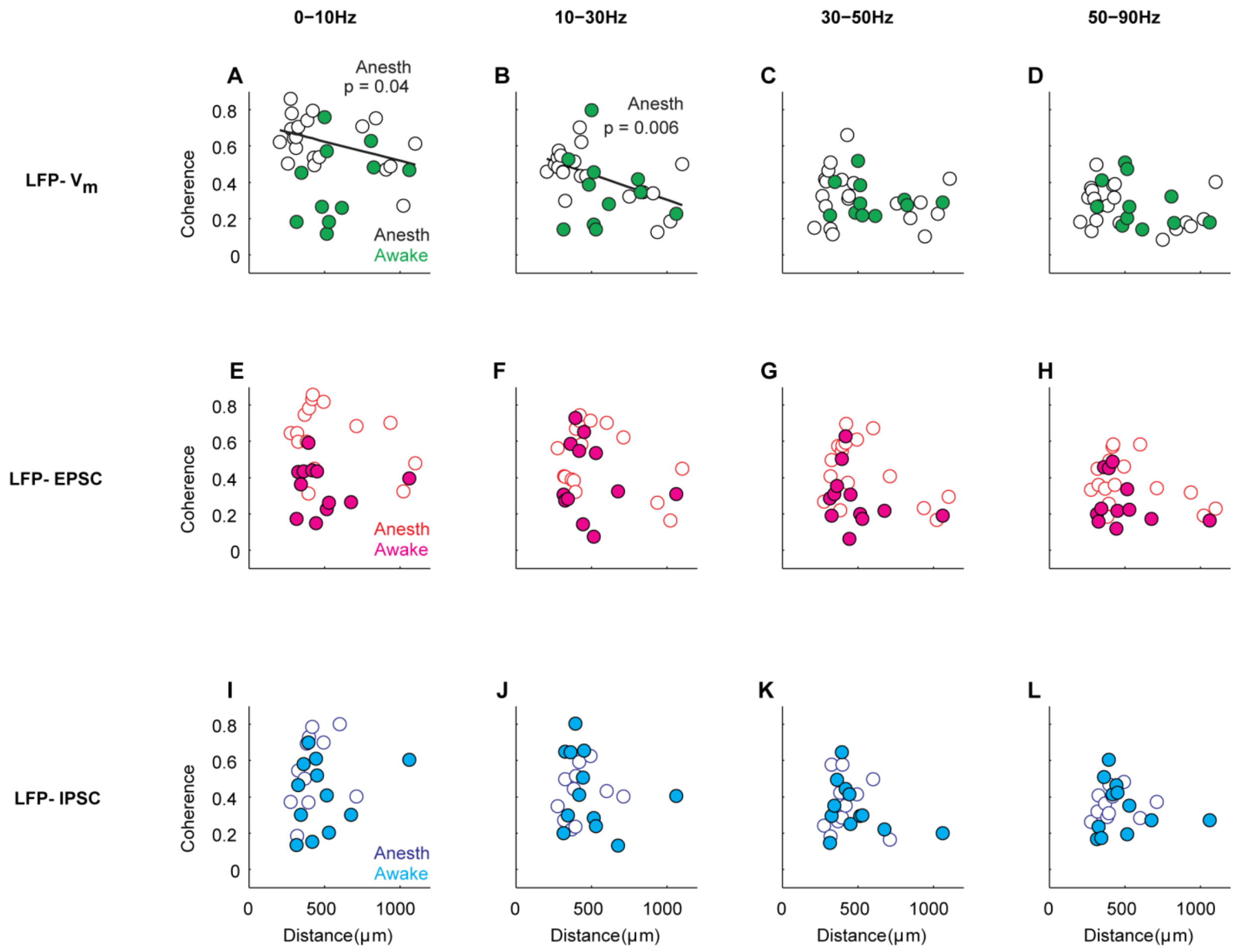

FIGURE S4 (Related to Figures 2 and 3). Coherence as a function of frequency band and separation distance.

A-D. Stimulus-evoked LFP-V_m coherence during anesthetized and awake states. Mean coherence plotted within band (see Figure S5 for coherence plots). **A**, Mean coherence < 10Hz. Significant correlation with distance during anesthesia. **B**, Coherence for 10 - 30Hz. Significant correlation with distance during anesthesia. **C**, Mean coherence from 30 - 50Hz. **D**, Mean coherence from 50 - 90Hz.

E-H. Stimulus-evoked LFP-EPSC coherence during anesthetized and awake states. Mean coherence plotted within band (see Figure S5 for coherence plots). **E**, Mean coherence < 10Hz. **F**, Coherence for 10 - 30Hz. **G**, Mean coherence from 30 - 50Hz. **H**, Mean coherence from 50 - 90Hz.

I-L. Stimulus-evoked LFP-V_m coherence during anesthetized and awake states. Mean coherence plotted within band (see Figure S5 for coherence plots). **I**, Mean coherence < 10Hz. **J**, Coherence for 10 - 30Hz. **K**, Mean coherence from 30 - 50Hz. **L**, Mean coherence from 50 - 90Hz.

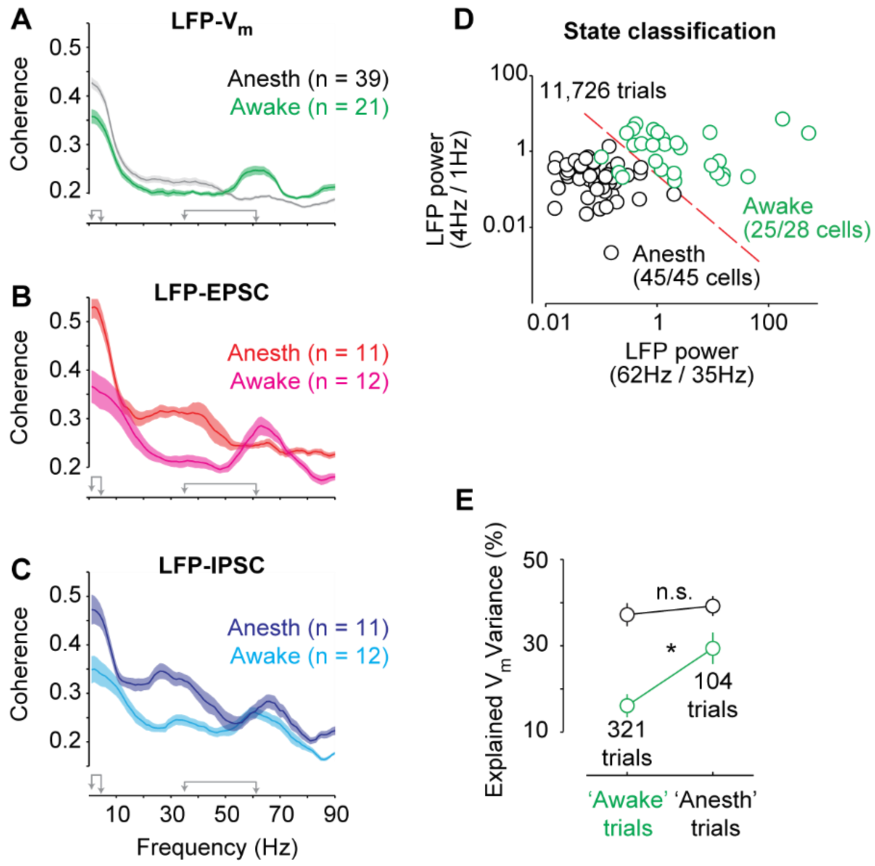

Figure S5 (Related to Figure 3). LFP features accurately distinguish anesthetized and awake states.

A. We wondered whether the differences in LFP coupling between anesthesia and wakefulness could arise from differences in the LFP signal itself. We observed clear differences in anesthetized and awake LFP spectral power, and LFP coherence with single neuron V_m across all paired recordings (anesthetized, black, n = 39; awake, green, n = 21). Arrows, 1-4 Hz and 35-62 Hz.

B. Spontaneous LFP-EPSC coherence (anesthetized, red, n = 11; awake, magenta, n = 12).

C. Spontaneous LFP-IPSC coherence (anesthetized, blue, n = 11; awake, cyan, n = 12).

D. We built a classifier that used LFP features to assign experiments to anesthetized and awake groups. This procedure segregated experiments with 96% accuracy (n = 73 recordings). Ratios of high-frequency (62 / 35 Hz) vs. low frequency (4 Hz / 1 Hz) LFP power accurately classify experimental conditions (n = 45/45 anesthetized recordings; n = 25/28 awake recordings correctly identified; 11,726 total trials). Power was calculated on individual trials, then averaged across trials (within recording) before taking log power ratios.

E. We then used the classifier to identify single trials where awake LFP activity was classified as anesthetized (and vice versa). On single trials where awake spontaneous activity displayed 'anesthetized' LFP power ratios (right, 104/425 individual trials), spontaneous V_m was in fact significantly more predictable than correctly classified awake trials (29.4 ± 3.6% vs. 16.1 ± 2.7%, $p < 0.01$ Wilcoxon rank sum test). No significant difference was observed across trial types during anesthesia.

These results show that cortical state fluctuations can be accurately segregated from the LFP alone, and confirm that cortical state fluctuations drive large trial-by-trial differences in the LFP and its coupling to single neurons.

Supplemental Experimental Procedures

Recordings

All procedures were in accordance with the Animals (Scientific Procedures) Act 1986, UK.

We made simultaneous LFP recordings and whole-cell patch-clamp recordings in L2/3 of mouse primary visual cortex (V1). LFP recordings were performed with glass patch pipettes filled with artificial cerebrospinal fluid (aCSF, 135mM NaCl, 5.4mM KCl, 5mM HEPES, 1mM $MgCl_2$ and1.8mM $CaCl_2$; pH 7.3).

Whole-cell patch-clamp recordings of V_m were performed with a K^+ gluconate internal solution (135mM potassium gluconate, 6mM KCl, 10mM HEPES, 4mM MgATP, 0.3mM Na_3GTP, 0.1mM EGTA and 8mM phosphocreatine, pH adjusted to 7.3 with KOH, 290–295 mOsM). For voltage clamp recordings of EPSCs (recorded near -80 mV) and IPSCs (recorded near +20 mV), potassium gluconate was substituted with 140mM cesium methanesulphonate, and QX-314 (0.5mM) and tetraethylammonium (TEA) (5mM) were included to block voltage-gated Na^+ and K^+ conductances.

The typical separation between our LFP and intracellular electrodes was ~0.5 mm (Anesthetized: 0.46 ± 0.02 mm; Awake: 0.53 ± 0.02 mm, mean ± sem), with a range of 0.2-1.1 mm, comparable to previous studies of LFP and subthreshold activity in awake cortex (Okun et al, 2010; Poulet and Petersen, 2008)

Most anesthetized recordings were performed under 0.5% – 1% isoflurane (Vm: 28/39 spontaneous, 18/21 stimulus-evoked; PSCs: 7/11) or urethane (1.5 g per kg, 10% w/v solution administered IP), plus chlorprothixene (10^{-5} mg per kg) in both cases. Awake recordings were performed in drug-free mice that were habituated to head-fixation over several days. Access resistance (in MΩ) was comparable across states, and was partially compensated during voltage clamp recordings (Anesth. current clamp: 40 ± 3; Awake current clamp: 38 ± 6; Anesth. voltage clamp: 36 ± 3 (15 ± 2 after compensation); Awake voltage clamp: 38 ± 4 (14 ± 2 after compensation).

Visual stimulation

Randomly chosen full contrast vertical black or white bars (9 degrees wide) were briefly flashed (100 ms duration), one at a time, in a randomly chosen spatial location on an LCD monitor spanning ± 45 degrees of visual space. Interstimulus intervals were 0.3-1.5 s. All recordings were performed in monocular V1 contralateral to the stimulated eye, with the receptive field of the recording at the center of the monitor (see Haider et al. 2013). Spontaneous activity was recorded in the presence of an isoluminant grey screen. Since both LFP and sub-threshold synaptic activity were elicited from large regions of visual space, we combined activity from all spatial locations for analysis of evoked activity.

Data processing

All signals were acquired at 20 kHz. LFP and PSCs were hardware filtered prior to digitization (LFP: 0.01-200 Hz; PSCs: < 2 kHz). The recordings of EPSCs and IPSCs were matched within neurons, performed sequentially, and with the same physical filter characteristics (of electrodes, series resistance, and capacitance) for paired EPSCs and IPSCs, across all individual experiments. Action potentials were digitally removed from V_m traces. The coherence between LFP and intracellular activity was computed using the Chronux toolbox (time-bandwidth NW=10, K=21 tapers) (Bokil et al., 2010).

Filter estimation

We first subtracted the mean across time from all LFP and V_m, EPSC, or IPSC traces. For the purposes of filter estimation during visual stimulation, we included the entire visual response (which lasted up to 1.5 second during anesthesia). We then estimated a filter constrained to be smooth in time via regularized linear regression. The algorithm works with the following quantities, where N is the number of samples in time:

- The LFP $\vec{u} = (u_1, \ldots, u_N)$ serving as input to the linear model.
- A trace (V_m / EPSC / IPSC) $\vec{y} = (y_1, \ldots, y_N)$ serving as output to be fitted.
- A set of time lags $\tau_1, \ldots, \tau_K$ spanning the filter $\vec{w} = \left(w_{\tau_1}, w_{\tau_2}, \ldots, w_{\tau_K}\right)$ to be estimated.

A so-called "lag matrix" $X = (\vec{x}_1, \ldots, \vec{x}_N)^T$ is formed by stacking vectors $\vec{x}_t$ which are formed by taking temporally extended snippets of the input $\vec{u}$ such that $\vec{x}_t = (u_{t-\tau_1}, \ldots u_{t-\tau_K})$.

A second matrix $C_{\sigma\lambda}$ – the prior covariance matrix - encodes the smoothness structure imposed on the optimal filter $\vec{w}_{\sigma\lambda}$, which – within the framework of regularized linear regression – is estimated as

$$\vec{w}_{\sigma\lambda} = (C_{\sigma\lambda}^{-1} + X^T X)^{-1} X^T \vec{y} \qquad (1)$$

The regularization structure is parameterized by a pair of numbers, the prior variance σ^2 and the smoothness λ, such that the prior covariance between filter entries w_i and w_j is given by $C_{\sigma\lambda}(w_i, w_j) = \sigma^2 \times \rho(w_i, w_j)$ where $\rho(w_i, w_j)$ is the prior correlation between filter weights w_i and w_j .

We chose an exponential correlation function in time, resulting in the following formula for the correlation:

$$\rho(w_i, w_j) = exp\left(-\frac{|\tau_i - \tau_j|}{\lambda}\right), \qquad (2)$$

where τ_i, τ_j are the filter lags of the weights.

When λ is small, the correlation of filter points nearby in time is also small. For example, this means that the filter value w_1 at time-lag τ_i=1 ms is largely independent from the value w_2 at a nearby point in time, such as τ_j=2 ms. Thus, a small λ captures our assumptions that *a priori* the filter $\vec{w}$ shows little smoothness in time.

However, when λ is large, then the correlation of nearby filter points is also large, meaning that *a priori* the filter values w_i and w_j should be similar, resulting in a temporal filter that looks smooth in time. Assuming that filters have a certain smoothness in time is reasonable, since single neurons and populations cannot respond arbitrarily fast. Furthermore, the smoothness prior prevents the algorithm from overfitting high-frequency noise which contains no signal (Suppl. Fig. 1).

We estimated the optimal hyperparameters $(\sigma, \lambda)_{opt}$ by performing gradient descent on the 10-fold cross-validation error. A given dataset is split into 10 disjoint parts, then filters are trained on 9 parts with the current prior covariance matrix $C_{\sigma\lambda}$. The estimated filter is used to predict the response on the omitted (test) set. The gradient is computed, iterated for all 10 parts, and all 10 gradients and errors are summed to search for the optimal hyperparameters $(\sigma, \lambda)_{opt}$ at which the cross-validation error is minimal. At that minimum, the trade-off between capturing the signal and avoiding overfitting to the noise is optimal.

The optimal hyperparameters averaged across cells and conditions were σ_{Vm} = 1.1, λ_{Vm} = 55 ms; σ_{EPSC} = 1.6, λ_{EPSC} = 50 ms; σ_{IPSC} = 3.2, λ_{IPSC} = 60 ms. The population of all filters is shown in Figs. 1 and 2 (mean ± SEM).

We did not use a separate test set that remained untouched throughout the analysis; a final test set is usually only employed when doing model comparisons, to determine which model performs best. We did not ask that question – we only used a single model.

We found the fitted hyperparameters to be similar across subsets of the data in the same condition. More specifically, when we determined the best hyperparameters for each of the 10 held-out sets, we found that they lie close to each other, strongly suggesting that no overfitting occurred.

In general, the risk of overfitting the hyperparameters is small because within each condition (e.g. spontaneous LFP-Vm, awake) we only fit two quantities, the variance σ^2 and the smoothness λ to the entire data set: tens of neurons with thousands of individual trials. When a vast amount of data is available to constrain just 2 numbers, the risk of overfitting is negligible.

Role of Autocorrelations

Slow autocorrelations of the LFP are the dominating factor in determining the shape of cross correlations. To obtain the underlying coupling filters, we need to correct for this autocorrelation. This correction, however, is only possible through regularization: there is too little energy at high frequencies in the autocorrelation, and without regularization this leads to noisy estimates.

To see this, consider first the case without regularization:

$$\vec{w}_{\text{unregularized}} = (X^T X)^{-1} X^T \vec{y} \quad (3)$$

where X is the LFP lag matrix, whose row vectors consist of time-shifted LFP traces. The matrix $X^T X$ is the autocorrelation matrix of the LFP, and $X^T \vec{y}$ is the cross-correlation of LFP with the signal of interest, $\vec{y}$.

To see the role of different frequencies in this estimation, let's turn to Fourier space – here we estimate the Fourier transform of the coupling filter by dividing the Fourier transform of the cross-correlation $X^T \vec{y}$ by the Fourier transform of the autocorrelation $X^T X$. If the latter is dominated by slow frequencies, its energy at high frequencies is small. Dividing by small and noisy quantities yields large and even noisier quantities.

This is indeed what we found when we followed this procedure: the filter estimated from unregularized linear regression is noisy, and does not recover ground-truth coupling because of high frequency noise (Fig. S2B). This reasoning reflects the fact that the autocorrelation contains little power at high frequencies and has its largest power at low frequencies.

The key contribution of the regularization procedure (Eq. 1) is to add just about enough energy $C_{\sigma\lambda}^{-1}$ at different frequencies of the autocorrelation function to reduce the impact of the noise when dividing by small noisy quantities. The regularization procedure (Eq. 1) is equivalent to filtering the cross-correlation $X^T \vec{y}$ by a filter F = $(C_{\sigma\lambda}^{-1} + X^T X)^{-1}$. But the filter F is not just any highpass filter: it is the optimal filter. We find it through a linear model and its associated squared error, which serves as a cost function and depends on hyperparameters σ, λ. If we only used the raw cross-correlation, we would have no such principled method of determining the frequency characteristics of the optimal filter for each particular condition.

References

Bokil, H., Andrews, P., Kulkarni, J.E., Mehta, S., and Mitra, P.P. (2010). Chronux: a platform for analyzing neural signals. J Neurosci Methods *192*, 146-151.
